# HLA-B57 micropolymorphism defines the sequence and conformational breadth of the immunopeptidome

**DOI:** 10.1038/s41467-018-07109-w

**Published:** 2018-11-08

**Authors:** Patricia T. Illing, Phillip Pymm, Nathan P. Croft, Hugo G. Hilton, Vladimir Jojic, Alex S. Han, Juan L. Mendoza, Nicole A. Mifsud, Nadine L. Dudek, James McCluskey, Peter Parham, Jamie Rossjohn, Julian P. Vivian, Anthony W. Purcell

**Affiliations:** 1 Infection and Immunity Program and Department of Biochemistry and Molecular Biology, Monash Biomedicine Discovery Institute, Monash University, Clayton, VIC 3800 Australia; 2 Australian Research Council Centre of Excellence for Advanced Molecular Imaging, Monash University, Clayton, VIC 3800 Australia; 3 Departments of Structural Biology and Microbiology & Immunology, School of Medicine, Stanford University, Stanford, 94305 CA USA; 4 Calico Life Sciences LLC, South San Francisco, 94080 CA USA; 5 Department of Genetics, School of Medicine, Stanford University, Stanford, 94305 CA USA; 6 Department of Molecular and Cellular Physiology, School of Medicine, Stanford University, Stanford, 94305 CA USA; 7 Institute for Molecular Engineering and Department of Biochemistry & Molecular Biology, University of Chicago, Chicago, 60637 IL USA; 8 Department of Microbiology and Immunology, Peter Doherty Institute for Infection and Immunity, University of Melbourne, Parkville, VIC 3010 Australia; 9 Institute of Infection and Immunity, Cardiff University School of Medicine, Heath Park, Cardiff, CF14 4XN UK

## Abstract

Immunophenotypic differences between closely related human leukocyte antigen (HLA) alleles have been associated with divergent clinical outcomes in infection, autoimmunity, transplantation and drug hypersensitivity. Here we explore the impact of micropolymorphism on peptide antigen presentation by three closely related HLA molecules, HLA-B*57:01, HLA-B*57:03 and HLA-B*58:01, that are differentially associated with the HIV elite controller phenotype and adverse drug reactions. For each allotype, we mine HLA ligand data sets derived from the same parental cell proteome to define qualitative differences in peptide presentation using classical peptide binding motifs and an unbiased statistical approach. The peptide repertoires show marked qualitative overlap, with 982 peptides presented by all allomorphs. However, differences in peptide abundance, HLA-peptide stability, and HLA-bound conformation demonstrate that HLA micropolymorphism impacts more than simply the range of peptide ligands. These differences provide grounds for distinct immune reactivity and insights into the capacity of micropolymorphism to diversify immune outcomes.

## Abstract (web summary)

Human leukocyte antigens (HLA) are multi-allelic and polymorphic genes that present antigens to immune cells for inducing protective immunity. Here, using systems biology and structural approaches, the authors show that micropolymorphism of three HLA has effects beyond the modulation of antigen diversity.

## Introduction

The human leucocyte antigen (HLA) molecules, encoded by the major histocompatibility complex (MHC) region of the genome, are cell surface glycoproteins responsible for the presentation of both endogenous and exogenously derived peptide antigens for immune surveillance. The introduction of novel complexes into this array, such as those containing peptides derived from invading pathogens, stimulates immune responses against infected cells. The genes encoding the HLA molecules (*HLA-A*, *-B* and *-C* for the classical HLA class I molecules, and *HLA-DP*, *-DQ* and *-DR* for the HLA class II molecules) are the most polymorphic of the human genome, with *HLA-B* alone possessing over 3000 functional allomorphs^1^. Sequence diversity in HLA class I molecules ranges from micropolymorphisms, which comprise just a few amino acids, to differences of more than 30 amino acids in more distantly related allomorphs. Peptides bind to HLA molecules via interactions between the side chains of anchor residues of the peptide and pockets within the antigen-binding cleft. In the HLA class I molecules, these pockets are denoted A–F, and a large part of their landscape is determined by polymorphic amino acid residues. These polymorphisms alter the stereo- and electrochemical environment of the pockets, dictating their ability to accommodate different peptide side chains thereby influencing the nature and quantity of peptides that are bound by a given HLA allomorph^2–4^. The nature of the peptide anchor residues accommodated by a particular HLA molecule is often referred to as the peptide-binding motif. Polymorphism further shapes the peptide array via impacting interactions with chaperones such as tapasin, which modulates peptide selection during peptide loading in the endoplasmic reticulum, biasing the peptide repertoire towards more stable ligands^5,6^.

Strikingly, polymorphism at a single amino acid in the antigen-binding cleft can cause divergent immune reactivity in many clinical scenarios. For example, ankylosing spondylitis is associated with some, but not all, HLA-B27 family members; for instance, HLA-B*27:02, -B*27:03, -B*27:04 and -B*27:05 confer risk, whilst micropolymorphic family members HLA-B*27:06 and -B*27:09 do not (reviewed^7^). Although the differential association of HLA-B27 allomorphs with ankylosing spondylitis has long been thought to be directly related to differences in ligand-binding characteristics, our recent studies have challenged this hypothesis, and show a more quantitative impact of micropolymorphism on the immunopeptidome rather than merely effects on ligand binding^8,9^. Similarly, abacavir hypersensitivity syndrome (AHS), a severe systemic hypersensitivity reaction to the antiretroviral drug abacavir and drug-induced liver injury mediated by the antibiotic flucloxacillin, are associated with HLA-B*57:01^10,11^, whilst the closely related HLA-B*57:03, containing two amino acid substitutions in the antigen-binding cleft shows no association. Similarly, HLA-B*58:01, possessing four substitutions in the antigen-binding cleft, shows no association with AHS^12^, but is instead strongly associated with allopurinol hypersensitivity^13^. It has been proposed that associations with adverse drug reactions are due to the unique ability of the associated HLA class I allomorph to present antigenic ligands, whether they be self-peptides, drug-modified peptides, or directly presented small-molecule drugs/metabolites^14^. However, whilst this view stands true for abacavir, which is uniquely accommodated within the antigen-binding cleft of HLA-B*57:01 in the vicinity of residues that are polymorphic between HLA-B*57:03 and HLA-B*58:01^15,16^, it may be too simplistic in the context of peptide presentation. For example, altered presentation of the same peptides by micropolymorphic allomorphs has been reported to impact immunogenicity and immunodominance hierarchies in the HLA-B35 family through altered plasticity and binding kinetics^17,18^, whilst distinct conformations of identical ligands presented by members of the HLA-B7 family are proposed to favour distinct escape mutations in human immunodeficiency virus (HIV)^19^. Equally, a single residue can delineate tapasin dependence for peptide loading and define the susceptibility of an HLA molecule to viral interference with the peptide loading pathway^5,20^.

HLA-B57 family members are renowned for their association with the elite controller phenotype of HIV-infected individuals^21,22^. The protective effect of HLA-B57 is hypothesised to be due to more efficient presentation of immunogenic HIV peptides to antiviral cytotoxic T lymphocytes than by non-protective HLA variants (possessing disparate peptide-binding properties). However, despite the near-identical nature of the previously described HLA-ligand-binding motif within this family, a protective hierarchy is still evident and distinctions in T-cell response to HIV epitopes are manifest^23^. Moreover, despite similar modes of presentation by HLA-B*57:01 and HLA-B*57:03, the immunodominant HIV-Gag-derived peptide KAFSPEVIPMF stimulates divergent T-cell responses in the context of these two allomorphs, with HLA-B*57:01 presentation generating a T-cell response able to recognise escape variants^24,25^.

These examples show the capacity for minor changes of the antigen-binding cleft to have marked effects on immune response and generate an impetus to understand more broadly the complexities of antigen presentation within the HLA-B57 family. HLA-B*57:01, HLA-B*57:03 and HLA-B*58:01 are micropolymorphic HLA allotypes of the HLA-B17 serotype. Polymorphism within these allotypes is focussed on regions of the antigen-binding cleft (cleft polymorphisms in comparison to HLA-B*57:01: Asp114Asn and Ser116Tyr in HLA-B*57:03, and Met45Thr, Ala46Glu, Val97Arg and Val103Leu in HLA-B*58:01). Notably, residues 97, 114 and 116 contribute to the E pocket of the antigen-binding cleft, whilst residue 116 also contributes to the F pocket. These are key locations of interaction between peptide ligands and the HLA heavy chain, with the F pocket accommodating the C-terminal anchor residue (PΩ), and the E pocket interacting with PΩ-2. The use of mass spectrometry to resolve binding preferences of single HLA allotypes is well established, and allows comparison of both micropolymorphic and distantly related molecules^8,26^.

Here we utilised a large database of constitutive peptide ligands isolated from HLA-B*57:01, HLA-B*57:03 and HLA-B*58:01^15^, to map micropolymorphism-dependent changes in the HLA peptide repertoire. Although speculated upon in many studies^27,28^, our study formally shows that subtle differences in primary and secondary anchor preferences of each allomorph correlate with altered stability of the respective HLA-peptide (pHLA) complexes and altered conformation of common peptides within the antigen-binding cleft. The ability to resolve these key differences is crucial to understanding altered disease outcomes between individuals having closely related HLA molecules that can represent ‘taboo mismatches’ in clinical transplantation^28,29^.

## Results

### Qualitative resolution of the HLA peptide-binding motifs

**Fig. 1:**
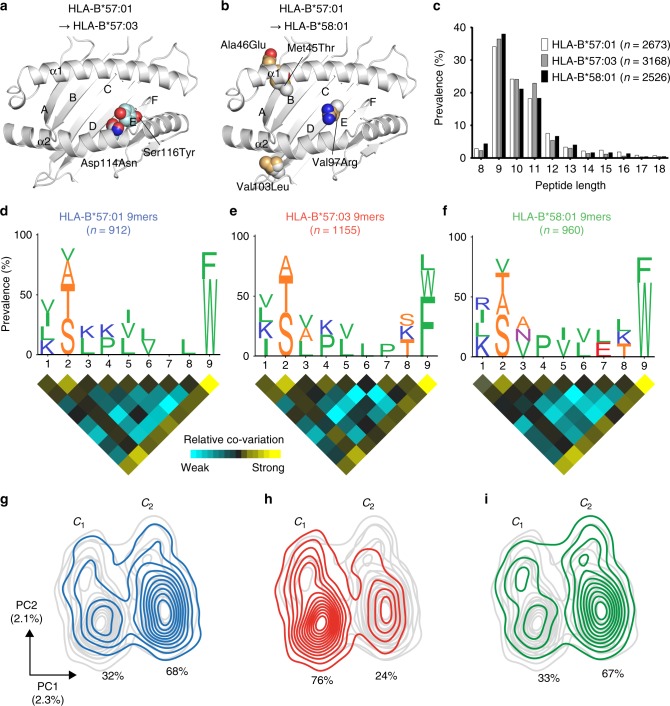
HLA-B*57:01, HLA-B*57:03 and HLA-B*58:01 sample peptides with similar physicochemical properties, but with different biases at the C terminus. **a**, **b** Ribbon diagrams depicting the peptide-binding grooves of HLA-B*57:03 and HLA-B*58:01 compared to HLA-B*57:01 (polymorphic residues are shown as space-filled models). α1 and α2 helices and the positions of peptide-binding pockets A–F are shown. **c** Length distributions of HLA-B*57:01 (clear bars), HLA-B*57:03 (grey bars) and HLA-B*58:01 (black bars) ligands across 8–18mers. **d**–**f** Sequence motifs of 9mers for each allomorph with accompanying co-variation heat maps, highlighting pairs of positions that are coupled. Amino acids are represented by single-letter code with height scaled to prevalence and colour representing small (orange), hydrophobic (green), polar (magenta), negatively charged (red) and positively charged (blue) residues. Only amino acids present with 10% or greater prevalence are depicted. *n* is the number of 9mer peptides within the data set. For the heat maps, square colour reflects the relative co-variation at, or between, each position in the peptide (aqua indicates weak co-variation and yellow indicates strong co-variation). **g**–**i** Principal component analysis (PCA) density plots showing the distribution of 9mer peptides across PC1 and PC2 for HLA-B*57:01 (**g**), HLA-B*57:03 (**h**) and HLA-B*58:01 (**i**). Coloured lines represent the named allomorph (HLA-B*57:01—blue, HLA-B*57:03—red and HLA-B*58:01—green) overlaid on the distribution of the remaining two allomorphs (grey lines). Two major clusters (c_1_ and c_2_) were defined using *k*-means clustering (*k* = 2) with percentage of 9mers within each cluster shown. Variation of PC1 and PC2 is shown in parentheses

To measure the impact of micropolymorphism on peptide presentation by HLA-B*57:01, HLA-B*57:03 and HLA-B*58:01 (cleft polymorphism locations depicted in Fig. 1a, b, described as changes in comparison to HLA-B*57:01 for B*57:03/B*58:01 throughout the manuscript) we interrogated large monoallelic ligand data sets (>2500 non-redundant peptide sequences) generated by isolation of naturally processed and presented HLA class I-bound peptides from individually transfected class I reduced (C1R) cells^15^. Of note, derivation from the same parental cell line ensured differences in peptide repertoire were attributable to differences in determinant selection by the HLA allotypes and not the source proteome or polymorphisms within antigen-processing machinery.

Consistent with previously reported HLA class I ligands, peptides bound to the three HLA-B allotypes were predominantly 9–11 residues in length with a preference for nonamers (Fig. 1c, Supplementary Data 1). Sequence motifs, generated from non-redundant lists of all 9–11mers depict specific amino acid preferences at each position of the peptide ligand. These preferences are very similar for the three allotypes, which display bias for Ser (S), Thr (T), Ala (A) and to a lesser extent Val (V) at P2, and aromatic residues at the C terminus (PΩ) (Fig. 1d–f, Supplementary Fig. 1a–c and 2a–c). Notably, whilst Trp (W) was the most prevalent PΩ residue for HLA-B*57:01 and HLA-B*58:01 ligands (62–80% of 9–11mers), HLA-B*57:03-bound peptides showed a higher prevalence of Phe (F) (43–50% Phe compared to 22–34% Trp across 9–11mers) (Supplementary Table 1, Fig. 1d–f, Supplementary Fig. 1a–c and 2a–c). In addition to traditional motif analysis, we also performed an unbiased statistical analysis of the allomorph-specific peptidomes using co-variation analysis (Fig. 1d–f). The P2 and PΩ preferences were observed to exist as conserved pairings within peptides with Ser at P2 strongly paired with PΩ Trp (HLA-B*57:01 and HLA-B*58:01) or Phe (HLA-B*57:03) in 9mers consistent with their nature as primary anchors (Supplementary Fig. 3).

In order to more deeply probe differences in ligand-binding characteristics, the physicochemical properties of each amino acid residue were used to independently compare the source of variation between the peptide repertoires. Each bound peptide was defined by a set of four parameters (in addition to 20 amino acid identity parameters) at each amino acid position: molecular weight, surface area, hydropathy index and isoelectric point. Consideration of broader physicochemical properties can capture similarities among amino acids that may be missed using traditional motif analysis^30^. Peptides of 9–11 residues in length that formed ligands of the three allotypes were then independently subjected to principal component analysis (PCA). For the three peptide lengths, the HLA ligands of each allotype were distributed asymmetrically across two distinct clusters defined by PC1 and PC2 (although further substructure was also observed for 9 and 11mers [Supplementary Fig. 4 and 5]). Although ligands mapped to both clusters for all variants, suggesting the potential to sample peptides of similar physicochemical properties, the bias of peptides between the clusters was inverted for HLA-B*57:03 relative to HLA-B*57:01 and -B*58:01 (Fig. 1g–i, Supplementary Fig. 1d–f and 2d–f). Parameters distinguishing these clusters include features of PΩ, reflected by the enrichment of Phe in cluster 1 (c_1_) and Trp in cluster 2 (c_2_) of the 9mer PCA plots, demonstrating this location is the main point of difference among the repertoires (Supplementary Fig. 6 and 7). Features of P1 were major contributors to PC2 (and PC1 for 10 and 11mers), and distributed peptides within these major clusters in a similar fashion for all allotypes. In addition to the amino acid identity, the PCA provides additional insights into ligand selection by the different allomorphs. Importantly, a major driver of the PCA was the amino acid surface area at PΩ, which was not the case at P2 where hydropathy index was the most important property. This pattern was true for each of the three allotypes and was independent of peptide length.

**Fig. 2:**
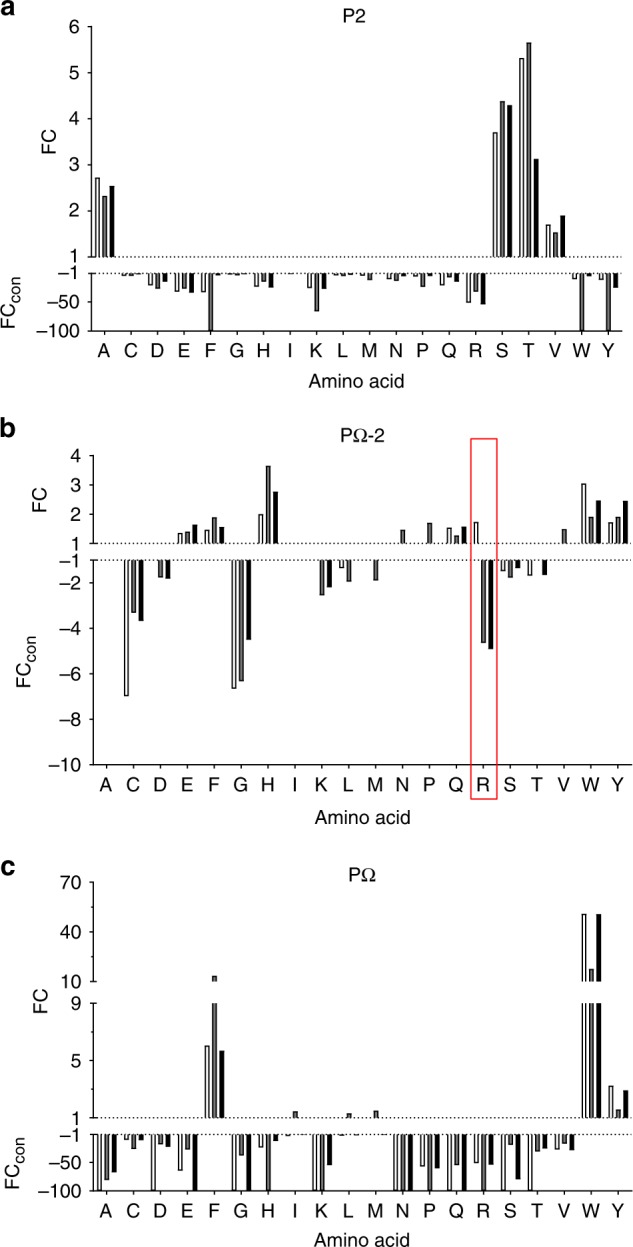
Enrichment/diminution of residues at P2, PΩ-2 and PΩ of HLA-B57 family ligands distinguish primary and secondary anchor sites. The enrichment of specific residues at P2 (**a**), PΩ-2 (**b**) and PΩ (**c**) of all 9 residue peptide binders of HLA-B*57:01 (clear bars, 912 peptides), HLA-B*57:03 (grey bars, 1155 peptides) and HLA-B*58:01 (black bars, 960 peptides) identified by LC-MS/MS relative to amino acid frequencies in the human proteome. Deviations in prevalence from the human proteome are depicted as either a fold change (FC, for amino acids at higher prevalence than in the human proteome) or a converted FC (FC_con_ = −1/FC, for amino acids present at lower prevalence than in the human proteome), as determined using iceLogo v1.2 stand-alone software^31^ using the static reference method (reference set *Homo sapiens* Swiss-Prot means, *p* < 0.05). FC > 1 indicates enrichment, FC_con_ < −1 indicates disfavoured residues, −100 indicates absence. In **b** the unique enrichment of Arg(R) at PΩ-2 by HLA-B*57:01 is highlighted by the red box

In addition to the main anchor residues, HLA-B*57:01 ligands showed a higher Arg (R), and to a lesser extent Lys (K), prevalence at PΩ-2 (i.e. P7, P8 and P9 of 9mer, 10mer and 11mer peptides, respectively), whilst HLA-B*58:01 ligands displayed greater PΩ-2 Glu (E) (Fig. 1d–f, Supplementary Fig. 1a–c and 2a–c, Supplementary Table 2). We therefore hypothesised that PΩ-2 is a secondary anchor site, shaped by polymorphic residues of the E pocket (Fig. 1a, b). Consequently, to probe the nature of the anchor sites more stringently we assessed enrichment of amino acid use at a particular location relative to global prevalence in the human proteome using iceLogo software^31^ (iceLogo v1.2, static reference method, *Homo sapiens* Swiss-prot means, *p* < 0.05). Enrichment was depicted as a fold change (FC) in prevalence at primary anchor locations P2 and PΩ, and the potential secondary anchor site PΩ-2, for 9mer peptides (Fig. 2). At both P2 and PΩ, strong enrichment of a small subset of amino acids (Ala, Ser, Thr and Val for P2 and Phe, Trp and Tyr for PΩ) was displayed whilst other amino acids were disfavoured (i.e. less prevalent than in the human proteome, depicted as converted FC [FC_con_]). This was particularly evident at PΩ, although HLA-B*57:03 showed some enrichment of Leu, Ile and Met in addition to aromatic residues (Fig. 2c). In contrast with the primary anchor sites, no amino acids were strongly disfavoured at PΩ-2. However, enrichment of Arg was a distinct feature of HLA-B*57:01 (FC 1.73), compared to HLA-B*57:03 (FC_con_ −4.65) and HLA-B*58:01 (FC_con_ −4.92) (Fig. 2b, red box). Lys at PΩ-2, though not significantly enriched for HLA-B*57:01 (FC 1.07, *p* > 0.05), was disfavoured by HLA-B*57:03 (FC_con_ −2.55) and B*58:01 (FC_con_ −2.20). Although most prevalent in the repertoire of HLA-B*58:01, Glu was enriched at PΩ-2 for all allotypes (FC 1.36–1.65). Pro showed enrichment at PΩ-2 for HLA-B*57:03 alone (FC 1.71), and was present in >10% of 9mer ligands of this allomorph, however this was not the case for 10 and 11mer peptides (Figs. 1d–f and 2b, Supplementary Fig. 1, 2). Collectively, these data resolve three distinct peptide-binding motifs based on the physicochemical properties and amino acid occupancy at different positions of the bound peptide ligand.

### Quantitative differences in presentation of common peptides

As anticipated there is a large overlap in peptide ligands presented with 982 peptides (by sequence alone) common to all three HLA-B allotypes (26–33% of the allotypic repertoire), increasing to 1361–1546 (38–51%) between allotype pairs (Supplementary Data 2). During tandem mass spectrometry (MS/MS) analysis, high quality of sequencing data was achieved by limiting fragmentation to the 30 most abundant species observed per second^15^. This selection resulted in the potential to miss less-abundant ions, especially in chromatographic regions of high complexity. Thus, it is likely that some of the peptides identified as unique to a particular allotype by liquid chromatography (LC)-MS/MS-based peptide identification were present at low concentrations in eluates from the other allotypes and as such failed to be selected for MS/MS.

Targeted LC-MS techniques such as multiple reaction monitoring (MRM) have been used to detect both high and low abundance ligands in highly complex samples eluted from the MHC and allow comparison of abundance^32,33^. Therefore, we designed an LC-MRM-MS approach to assess whether peptides were identified as unique to a particular allotype due to sampling issues rather than binding specificity. To this purpose, we generated a representative list containing 60 native peptides from those identified by MS/MS in biological replicate experiments for a given allotype and could be identified in eluates from 10^8^ cells of at least one allotype. These peptides were selected from each of the following categories: unique to an allotype, common to all allotypes or common to two of the three allotypes. Three new independent biological replicates were analysed per allotype by LC-MRM-MS and individual peptide relative abundance determined across samples.

**Fig. 3:**
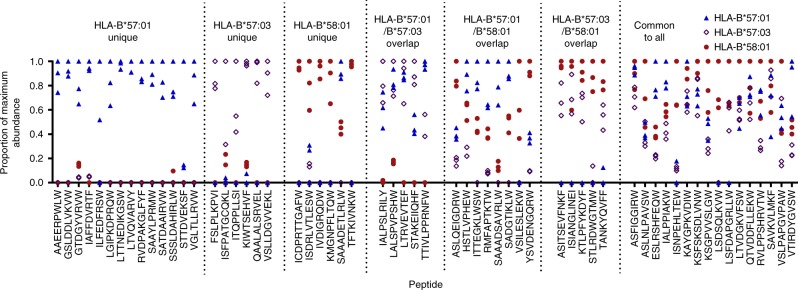
MRM analysis reveals quantitative differences in peptide presentation and suggests greater overlap in peptide repertoire. Relative abundance of peptide ligands bound to HLA-B*57:01 (blue triangles), HLA-B*57:03 (open purple diamonds) or HLA-B*58:01 (red circles) as detected by LC-MRM-MS. Peptides are categorised based on previous detection by LC-MS/MS as either binding a single HLA allotype (unique), two HLA allotypes (overlap) or all three HLA allotypes (common). On peptide detection the combined peak area for all MRM transitions, normalised against co-immunoprecipitated β_2_m, was used to calculate relative abundance. Abundances are shown as a proportion of the maximum normalised peak area detected for the peptide across all experiments. Data are shown for three biological replicate experiments per allotype

Using this sampling independent approach 5/17, 7/28 and 6/25 (3/17, 7/28 and 5/25 in multiple replicates) of the peptides not previously identified in eluates from HLA-B*57:01, HLA-B*57:03 and HLA-B*58:01 respectively were detected, generally at considerably lower relative abundance than for the allotypes for which they were initially described as ligands (Fig. 3), supporting the rationale for this targeted approach. Indeed, although only identified with a deaminated Arg at P9 in the immunopeptidome of HLA-B*57:01 by LC-MS/MS (confidence 95, >5% false discovery rate (FDR), Supplementary Data 2), native SAAADETLRLW contributed most to the immunopeptidome of this allotype. Quantitative differences across the allotypes were observed for all peptides, even those that were consistently isolated from all three allotypes in the original non-targeted LC-MS/MS experiments, implying quantitative, as much as qualitative, differences distinguish the immunopeptidomes of these closely related allotypes.

### PΩ and PΩ-2 preferences correlate with pHLA stability

Given the differences in PΩ and PΩ-2 anchor preferences, we examined the impact of these residues on pHLA complex stability. To do so we chose two 9mers containing Arg/Lys at P7 (PΩ-2) that formed a natural part of the HLA-B*57:01 peptide repertoire, LSSPVTKSF and LTVQVARVY, and designed P7/9 variants, to utilise in thermal stability experiments. LSSPVTKSF was also identified in the repertoires of HLA-B*57:03 and HLA-B*58:01 by LC-MS/MS, and the structure of LSSPVTKSF in complex with HLA-B*57:01 has been published previously, showing a salt-bridge between P7Lys and Asp114 of the HLA-B*57:01 heavy chain (PDB 2RFX)^34^. LTVQVARVY was not detected in the repertoire of HLA-B*57:03 or HLA-B*58:01 (Fig. 3).

LTVQVARVY complexes were markedly more stable in the context of HLA-B*57:01 than HLA-B*57:03 (~9 °C difference in temperature for 50% unfold [*T*_m_], Table 1). Consistent with the enrichment of P7Arg by HLA-B*57:01 alone, the P7Gln mutation (chosen due to similar enrichment of this residue at P7 by all allotypes, Fig. 2b) reduced this difference to ~4 °C by increasing the stability of HLA-B*57:03 complexes and reducing the stability of HLA-B*57:01 complexes. In contrast, the substitution of the P9Tyr for Trp, which is more enriched at this location in ligands of both allotypes, improved the stability of both HLA-B*57:01 (+2.8 °C) and HLA-B*57:03 (+8.6 °C) complexes. The P7Gln mutation had less impact in the context of P9Trp (<2 °C) for both HLA-B*57:01 and HLA-B*57:03 but increased the stability of HLA-B*58:01 complexes (+4.6 °C), consistent with disfavoured P7Arg by HLA-B*58:01 (Fig. 2b).

**Table 1:** The impact of PΩ-2 and PΩ peptide residue substitutions on HLA-peptide complex thermal stability

Peptide sequence	HLA-B*57:01 *T*_m_ (°C)	PΩ-2 *Q*	PΩ *W*	X	HLA-B*57:03 *T*_m_ (°C)	PΩ-2 *Q*	PΩ *W*	X	HLA-B*58:01 *T*_m_ (°C)	PΩ-2 *Q*	PΩ *W*	X
LSSPVTKSF (wt)	70.6 (0.74)	−0. 2	**2.0**		69.2 (0.35)	0.4	**1.8**		69.3 (0.52)	0.9	**2.5**	
LSSPVTQSF	70.4 (0.26)	–	**2.7**		69.6 (0.11)^a^	–	**1.5**		70.2 (0.7)	–	**2.8**	
LSSPVTKSW	72.6 (0.41)	0.5	–	X	71.0 (0)	0.1	–	X	71.8 (0.61)	**1.2**	–	X
LSSPVTQSW	73.1 (0.18)^a^	–	–		71.1 (0.1)	–	–		73.0 (0.32)	–	–	
LTVQVARVY (wt)	69.4 (0.18)^a^	−2.3	**2.8**	X	60.6 (0.67)	**2.5**	**8.6**	X	N.D.	–	–	
LTVQVAQVY	67.1 (0.18)^a^	–	**3.9**		63.1 (0.14)	–	**7.8**		N.D.	–	–	
LTVQVARVW	72.2 (0.46)	−1.2	–	X	69.2 (0.09)	**1.7**	–	X	67.8 (1.12)	**4.6**	–	X
LTVQVAQVW	71.0 (0.48)^b^	–	–		70.9 (0.19)	–	–		72.4 (0.61)^c^	–	–	
SAAADETLRLW (wt)	67.5 (0.99)	–	–		61.3 (0.17)	–	–		64.4 (0.67)	–	–	

Thermal stability is reported as mean temperature for 50% unfold (*T*_m_), standard deviation is shown in parentheses. Values are calculated from duplicates at 1 mg mL^−1^ protein and 0.5 mg mL^−1^ protein (4 total) unless indicated otherwise. The impact of the PΩ-2 to *Q* and PΩ to *W* on *T*_m_ are shown where applicable (‘–’ is placed where not applicable), changes of magnitude > 1 °C are underlined (decreased stability) or in bold (increased stability). Column X denotes complexes for which structures were solved using X-ray crystallography

*ND* not determined due to poor HLA-peptide refold

^a^Values calculated from duplicates at 0.5 mg mL^−1^ protein alone

^b^Values calculated from duplicates at 0.8 and 0.5 mg mL^−1^ protein

^c^Values calculated from quadruplicates at 1 and 0.5 mg mL^−^^1^ protein

For pHLA containing LSSPVTKSF, the impacts of P7/P9 substitutions were less pronounced. For all allotypes mutation of the P7Lys to Gln had marginal impact on the *T*_m_ (<1 °C), although the P9Phe to Trp mutation showed a trend towards stabilising all complexes (+1.8–2.5 °C). Introduction of the P7Gln mutation also had minimal impact in the context of P9Trp mutation. The reduced influence of the P7Lys to Gln mutation is consistent with the weaker enrichment/diminution of P7Lys as compared to P7Arg displayed by each allotype (Fig. 2b). Similarly, all allotypes show less selective discrimination between Phe and Trp at P9 than between Tyr and Trp (Fig. 2c), consistent with a smaller impact of mutation of P9 to Trp in the context of LSSPVTKSF compared to LTVQVARVY.

SAAADETLRLW, detected in the repertoire of HLA-B*58:01 (MS/MS and MRM) and HLA-B*57:01 (MRM only) as described above, was also subject to thermal stability assays. The trend in thermal stability across the allomorphs (HLA-B*57:01 [67.5 °C] > HLA-B*58:01 [64.4 °C] > HLA-B*57:03 [61.3 °C], Table 1) correlated with the relative abundance detected in their repertoires by MRM (HLA-B*57:01 > HLA-B*58:01 > HLA-B*57:03, Fig. 3).

### Conformations of common ligands differ between allomorphs

To understand the structural effects of micropolymorphism between HLA-B*57:01, HLA-B*57:03 and HLA-B*58:01 and its relationship to peptide association, crystal structures in complex with the peptides LTVQVARVW, LTVQVARVY and LSSPVTKSW were determined to resolutions of 1.6–2.0 Å (data collection and refinement statistics summarised in Table 2). The high quality of the resultant models allowed for direct and reliable comparison of the structures (Supplementary Fig. 8). The three HLA molecules had similar overall tertiary structures for each peptide complex (root mean square deviation values ranging from 0.16 to 0.51 Å over Cα positions for residues 1–175) and there were no significant deviations in the secondary structure elements of the peptide-binding cleft (Supplementary Fig. 9). As such, the differential peptide-binding preferences were not due to any gross structural differences but rather to subtle differences in the architecture of the peptide-binding pockets.

**Table 2:** Data collection and refinement statistics

	HLA-B*57:01-LTVQVARVY	HLA-B*57:01-LTVQVARVW	HLA-B*57:01-LSSPVTKSW	HLA-B*57:03-LTVQVARVY	HLA-B*57:03- LTVQVARVW	HLA-B*57:03-LSSPVTKSW	HLA-B*58:01- LTVQVARVW	HLA-B*58:01-LSSPVTKSW
*Data collection statistics*								
Temperature (K)	100	100	100	100	100	100	100	100
X-ray source	MX2 Australian synchrotron	MX2 Australian synchrotron	MX2 Australian synchrotron	MX2 Australian synchrotron	MX2 Australian synchrotron	MX2 Australian synchrotron	MX2 Australian synchrotron	MX2 Australian synchrotron
Space group	*P*2_1_2_1_2_1_	*P*2_1_2_1_2_1_	*P*2_1_2_1_2_1_	*P*2_1_2_1_2_1_	*P*2_1_2_1_2_1_	*P*2_1_2_1_2_1_	*P*2_1_2_1_2_1_	*P*2_1_2_1_2_1_
Cell dimensions	*a* = 50.4, *b* = 81.5, *c* = 109.0	*a* = 50.7, *b* = 81.8, *c* = 109.3	*a* = 50.6, *b* = 81.6, *c* = 110.3	*a* = 50.8, *b* = 81.8, *c* = 109.5	*a* = 50.9, *b* = 81.9, *c* = 109.8	*a* = 50.6, *b* = 81.8, *c* = 110.0	*a* = 50.0 *b* = 81.9, *c* = 110.1	*a* = 50.7, *b* = 81.8, *c* = 109.7
Resolution (Å)	50–1.90 (2.00–1.90)	50–1.80 (1.90–1.80)	50–2.00 (2.11–2.00)	50–1.80 (1.90–1.80)	50–1.80 (1.90–1.80)	50–2.00 (2.11–2.00)	50–2.00 (2.11–2.00)	50–1.60 (1.69–1.60)
Total no. of observations	240 990 (34 763)	234 376 (34 331)	231 704 (33 624)	234 123 (33 600)	283 895 (40 374)	170 623 (24 847)	170 326 (24 582)	438 590 (60 847)
No. of unique observations	36 221 (5213)	42 942 (6172)	31 727 (4561)	42 970 (6161)	43 277 (6224)	31 614 (4559)	31 297 (4468)	60 877 (8687)
Multiplicity	6.7 (6.7)	5.5 (5.6)	7.3 (7.4)	5.4 (5.5)	6.6 (6.5)	5.4 (5.5)	5.4 (5.5)	7.2 (7.0)
Data completeness (%)	100 (100)	100 (100)	100 (100)	99.9 (99.7)	99.8 (99.4)	99.9 (100)	99.9 (100)	99.7 (98.7)
1/*σ*_I_	9.7 (3.1)	12.4 (3.7)	12.6 (3.2)	9.5 (3.4)	10.1 (2.7)	10.2 (3.7)	8.3 (2.8)	22.7 (4.3)
*R*_merge_^a^	0.16 (0.61)	0.10 (0.51)	0.16 (0.70)	0.14 (0.71)	0.14 (0.74)	0.14 (0.49)	0.18 (0.61)	0.058 (0.467)
*Refinement statistics*								
Non-hydrogen atoms								
Protein	3110	3110	3109	3119	3164	3112	3135	3165
Water	568	533	454	611	669	559	458	644
* R*_factor_ (%)^b^	17.5	16.5	17.4	15.7	16.7	16.9	17.3	17.3
* R*_free_ (%)^b^	21.5	20.7	20.9	19.6	20.4	20.2	21.4	19.9
r.m.s.d. from ideality								
Bond lengths (Å)	0.005	0.010	0.005	0.019	0.007	0.004	0.007	0.007
Bond angles (°)	0.858	1.336	0.882	1.85	1.060	0.833	1.100	1.048
Dihedrals (°)	13.8	13.8	13.9	14.5	13.5	13.1	13.6	14.0
Ramachandran plot								
Favoured regions (%)	98.7	98.7	98.4	98.4	98.7	98.4	98.7	98.2
Allowed regions (%)	1.3	1.3	1.6	1.6	1.3	1.6	1.3	1.8
*B*-factors (Å^2^)								
Average main chain	11.2	14.2	15.2	11.4	13.3	12.4	17.1	15.0
Average side chain	15.4	18.6	19.6	16.2	18.7	17.0	21.0	20.7
Average water	27.4	29.8	28.4	29.2	31.1	27.4	30.0	33.6

^a^*R*_merge_ = ∑_hkl_ ∑_j_ |*I*_hkl,j_ − < *I*_hkl_ > | / ∑_hkl_ ∑_j_ *I*_hkl,j_

^b^*R*_factor_ = ∑_hkl_‖*F*_o_| − |*F*_c_‖/∑_hkl_|*F*_o_| for all data excluding the 5% that comprised the *R*_free_ used for cross-validation

**Fig. 4:**
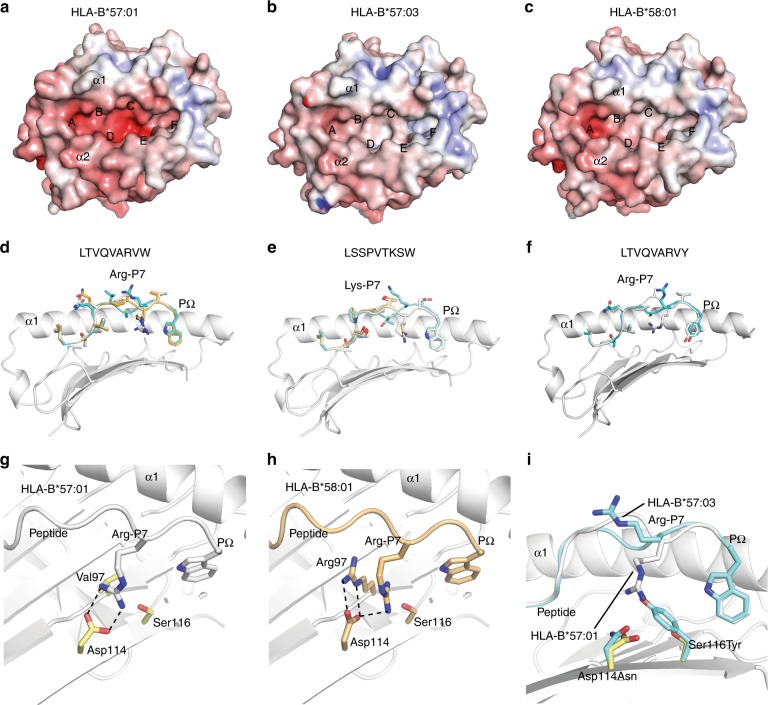
HLA-B*57:01, HLA-B*57:03 and HLA-B*58:01 present peptides in distinct conformations. **a**–**c** The electrostatic potential mapped to the surface of the structures of HLA-B*57:01, HLA-B*57:03 and HLA-B*58:01 respectively (red—electronegative, blue—electropositive). The α1 and α2 helices and the positions of peptide-binding pockets A–F are shown. **d**–**f** Superposition of the crystal structures of the LTVQVARVW, LSSPVTKSW and LTVQVARVY peptides (respectively) in complex with HLA-B*57:01 (grey), B*57:03 (cyan) and B*58:01 (orange). The α2 helix has been removed for clarity. **g** The interaction between the P7Arg (from the peptide LTVQVARVW) and Asp114 in HLA-B*57:01. **h** The interaction between the P7Arg (from the peptide LTVQVARVW) and Asp114 in HLA-B*58:01. **i** Superposition of the LTVQVARVW peptides in complex with HLA-B*57:01 (grey) and B*57:03 (cyan). The presence of the Ser116Tyr micropolymorphism (HLA-B*57:01–>HLA-B*57:03) prevents the binding of the P7Arg in the E pocket of HLA-B*57:03

The three HLA molecules are differentiated by polymorphisms distributed across the length of the peptide-binding groove (Fig. 1a, b). As a consequence of these substitutions the C, D, E and F pockets of HLA-B*57:01 were the deepest and most negatively charged of the three allomorphs (Fig. 4a). HLA-B*58:01 had the most shallow C and D pockets and B*57:03 the shallowest E and F pockets (Fig. 4b, c). Peripheral to the peptide-binding groove are further substitutions at positions Ala46Glu and Val103Leu (Fig. 1b) that subtly affect the structure of the β3-β4 and β5-β6 loops respectively (Supplementary Fig. 9). However, it should be noted that the location of these peripheral polymorphisms suggests that they are unlikely to directly influence T-cell receptor (TCR) recognition. These observations are consistent with the shared P2 anchor preference across the allomorphs, whilst the shallower F pocket of HLA-B*57:03 correlates with greater permissiveness for Phe and smaller non-aromatic anchors at PΩ (Fig. 2c) and the reduction in preference for high amino acid surface area at PΩ in the PCA. Whilst differences in F pocket architecture did not impact upon the conformation of Phe and Trp residues at PΩ, Tyr116 of HLA-B*57:03 restricted the accommodation of the hydroxyl group of PΩ Tyr, illustrated by a 1.4 Å shift of this group observed between HLA-B*57:01-LTVQVARVY and HLA-B*57:03-LTVQVARVY structures (Fig. 4f).

Notwithstanding the differences at the F pocket, the more striking differences in peptide conformation appear to be engendered by the respective D and E pocket environments. The depth and negative charge of the D and E pockets of HLA-B*57:01 accommodated the Arg and Lys residues at the PΩ-2 position of LTVQVARVW and LSSPVTKSW with the guanidino-head group of the Arg bound deeply within the E pocket, interacting with Asp114 (Fig. 4d, e, g). HLA-B*58:01 was similarly able to accommodate Arg and Lys residues within the E pocket, although the manner in which the Arg at PΩ-2 interacted with Asp114 differed. That is, in HLA-B*57:01-LTVQVARVW the PΩ-2 Arg formed a bi-dentate salt-bridge interaction with Asp114 (Fig. 4g), whilst in HLA-B*58:01-LTVQVARVW the PΩ-2 Arg side chain was twisted by 60° and the Asp114 side chain rotated 90° to accommodate the Val97Arg micropolymorphism (Fig. 4h). This altered the salt-bridge between the PΩ-2 Arg and Asp114 to a less favourable conformation in HLA-B*58:01, consistent with the reduced thermal stability of the complex (Fig. 4h, Table 1). In contrast, Arg and Lys were unable to be accommodated in the E pocket of HLA-B*57:03. Instead, due to the reduced E pocket volume caused by the Ser116Tyr substitution, these residues deviated by 9.7 Å and pointed out of the groove (Fig. 4d–f, i). The deviation at PΩ-2 was concomitant with a 5.3 Å shift and 180° rotation of the PΩ-3 residue to fill the C-pocket of HLA-B*57:03 (Fig. 4d–f). Overall, these structures showed that the buried polymorphisms generated minimal differences to the surface of the HLA molecule available to TCRs whilst engendering marked differences in the peptide surface landscape available for T-cell interaction.

### Reciprocal T-cell alloreactivity occurs between allotypes

**Fig. 5:**
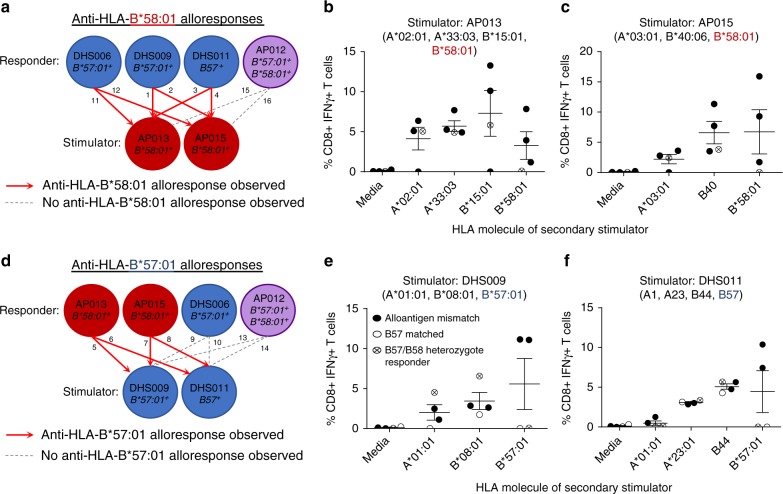
Reciprocal T-cell alloreactivity is observed between HLA-B*57:01 and HLA-B*58:01. Allogeneic MLRs were performed to measure the alloresponse to either HLA-B*57:01 or HLA-B*58:01 in healthy donors. Schematics show the responder/stimulator combinations utilised to stimulate outgrowth of anti-HLA-B*58:01 (**a**) and anti-HLA-B*57:01 (**d**) allo-specific T cells. Numbers correspond to the full details of each responder/stimulator combination provided in Supplementary Table 3. **b**, **c**, **e**, **f** IFNγ responses by CD8^+^ T cells of outgrown responders in a secondary stimulation utilising APCs expressing single HLA molecules of the primary stimulator as noted. Mean IFNγ response (±SEM) by all responders is shown for each HLA (calculated as IFNγ response minus background as indicated in Supplementary Table 3). Anti-HLA-B*58:01 alloreactivity was generated following stimulation with either AP013 (**b**) or AP015 (**c**) for allogenic mismatched responders (solid circles) but not for the HLA-B*57:01/B*58:01 heterozygote responder (crossed circle). Anti-HLA-B*57:01 alloreactivity was generated following stimulation with either DHS009 (**e**) or DHS011 (**f**) for allogenic mismatched responders (solid circles). Anti-HLA-B*57:01 alloreactivity was not observed with either HLA-B*57:01 matched (open circle) or a HLA-B*57:01/B*58:01 heterozygote responder (crossed circle). Note that absent responses to HLA-A*02:01 in **b** correspond to MLR 11, where responders/stimulators were matched for HLA-A*02:01

Vigorous T-cell alloresponses can be generated by a high degree of HLA class I mismatching between allogeneic individuals or as little as a single amino acid mismatch (e.g. across HLA-B44 allotypes)^28,35–37^. Here we examined whether the closely related alleles HLA-B*57:01 and HLA-B*58:01 are capable of eliciting either anti-HLA-B*58:01 (B*57:01 responder vs B*58:01 stimulator) or anti-HLA-B*57:01 (B*58:01 responder vs B*57:01 stimulator) CD8^+^ T-cell alloreactivity, which would support distinct presentation of the immunopeptidome. Experiments did not include HLA-B*57:03 due to a lack of availability of HLA-B*57:03^+^ donors who are rare in Caucasian populations^38,39^. A total of 16 unidirectional mixed lymphocyte reactions (MLRs) were performed utilising a combinatorial matrix incorporating six healthy individuals (Fig. 5a, d, Supplementary Table 3). Alloreactive T cells were expanded for 13 days, after which these bulk T-cell cultures were restimulated with a panel of B-lymphoblastoid cell lines (B-LCLs) expressing the mismatched stimulator HLA-A and -B alloantigens (Supplementary Table 3) to dissect their individual contribution (measured by interferon-gamma (IFNγ) production) to the overall alloresponse.

The first set of MLRs (1–4, 11, 12, 15 and 16; Fig. 5a, Supplementary Table 3) were designed to evaluate anti-HLA-B*58:01 T-cell alloreactivity between responders expressing HLA-B57 (DHS011), HLA-B*57:01 (DHS006 and DHS009) or both HLA-B*57:01 and HLA-B*58:01 (heterozygote, AP012), and stimulators expressing HLA-B*58:01 (AP013 and AP015). All HLA-A and -B mismatched alloantigens expressed by the stimulators, including HLA-B*58:01, generated alloreactive CD8^+^ T-cell responses. In contrast, no allo-specific CD8^+^ T cells were directed against HLA-B*58:01 in the heterozygote responder (Fig. 5b, c). The second set of MLRs (5–10, 13 and 14; Fig. 5d, Supplementary Table 3) were designed to evaluate anti-HLA-B*57:01 T-cell alloreactivity between responders expressing HLA-B*58:01 (AP013 and AP015), HLA-B*57:01 (matched, DHS006) or both HLA-B*57:01 and HLA-B*58:01 (heterozygote, AP012) and stimulators expressing HLA-B57 (DHS011) or HLA-B*57:01 (DHS009). Similar to the first set of MLRs, all HLA-A and -B mismatched alloantigens expressed by the stimulators generated alloreactive CD8^+^ T-cell responses, whilst no allo-specific CD8^+^ T cells were directed against HLA-B*57:01 by either the HLA-B*57:01/B*58:01 heterozygote or the HLA-B*57:01 matched responder (Fig. 5e, f). Thus, differences in self-peptide presentation by HLA-B*57:01 and HLA-B*58:01, not background proteome, generate alloreactivity between these related molecules.

## Discussion

In order to resolve the impacts of micropolymorphism on the peptide repertoire, we comprehensively analysed data sets comprising 2673 HLA-B*57:01-bound peptides, 3168 HLA-B*57:03-bound peptides and 2526 HLA-B*58:01-bound peptides isolated from monoallelic C1R transfectants^15^. Due to differences in the ionisation efficiencies of individual peptides, which precludes absolute peptide quantitation without the introduction of sequence-matched isotope-labelled standards, distribution of specific sequence features across the population of peptides was used to define and compare the peptide-binding motif of each allotype. The analysis was performed at three levels; first, at the level of amino acid preference individually for 9, 10 or 11mer peptide ligands; second, at the level of amino acid physical chemistry using a recently established PCA-based statistical analysis of the data; and finally, at the level of proteome enrichment. The majority of peptide ligands identified were 9–11 residues in length and possessed Ser, Thr or Ala at P2, and aromatic residues at their C terminus, consistent with investigations of HLA-B*57:01 and HLA-B*58:01 from other groups^26,40,41^. Of the three allotypes, the previously underexplored HLA-B*57:03 had the most distinctive binding preferences. Although length and P2 preferences were equivalent to the other allomorphs, preferences at PΩ differed, showing greater enrichment of Phe and greater sampling of ligands with smaller PΩ residues. Despite this, HLA-B*57:03, like HLA-B*57:01 and HLA-B*58:01, was more stable when in complex with peptides containing the bulky PΩTrp (compared to PΩTyr or Phe). The incongruity between the stabilisation effects conferred by Trp vs Phe at P9 and their prevalence in the repertoire of HLA-B*57:03 suggests an interplay between ligand availability (Trp and Phe constitute approximately 1 and 4% of the human proteome respectively) and complex stability in shaping the resultant peptide repertoire, which may be further influenced by interactions with the peptide-loading complex. Indeed, evidence of a hierarchy of tapasin dependence between these allomorphs (HLA-B*57:01 > HLA-B*58:01 > HLA-B*57:03)^42^ may indicate that HLA-B*57:03 can more readily escape the benefits of peptide editing within the peptide-loading complex, as suggested for other alleles^43^.

We further defined a secondary anchor site that modulated peptide affinity for the HLA molecule and distinguished the HLA-B*57:01 peptide-binding motif. Arg appeared at P7 of 9mers (and PΩ-2 of longer peptides) almost exclusively in the HLA-B*57:01 data set and correlated with stabilisation of HLA-B*57:01 complexes by PΩ-2 Arg. In contrast, PΩ-2 Arg negatively impacted the stability of HLA-B*57:03 and HLA-B*58:01 complexes. Structural analysis of bound peptide conformation showed a pronounced change in orientation of PΩ-2 Arg/Lys conferred by the Ser116Tyr polymorphism of HLA-B*57:03 that would markedly change the surface presented to T cells. More subtle changes were induced by the Val97Arg polymorphism in HLA-B*58:01. These observations strongly parallel differences in peptide presentation between micropolymorphic allotypes HLA-B*35:01 and HLA-B*35:08, in which a Leu156Arg substitution generates a secondary anchor site that improves the binding kinetics of peptides containing a negatively charged residue at P5. Of note, this results in distinct immunodominance hierarchies for human cytomegalovirus pp65 T-cell epitopes in HLA-B*35:01^+^ and HLA-B*35:08^+^ individuals^18^. In addition, the Leu156Arg polymorphism can alter peptide conformation and the plasticity of bound ligands, resulting in divergent T-cell responses to several Epstein Barr virus epitopes. Changes in T-cell responses were attributed to the adoption of different conformations within the antigen-binding cleft and/or the ability of the HLA-peptide complex to accommodate conformational change on TCR engagement^17,44,45^. Although HLA-B*57:01, HLA-B*57:03 and HLA-B*58:01 all associate with long-term non-progression of HIV-1 infection to acquired immunodeficiency syndrome^21,22^, differences in viral load between patients possessing different HLA-B57/58 alleles correlate with differential immunogenicity of identical peptide ligands^23^. The differences in peptide presentation described here provide grounds for this differential T-cell recognition of B57/B58-bound ligands. Indeed, HLA-B*57:01 and HLA-B*57:03 restricted presentation of the 11mer KAFSPEVIPMF HIV-Gag162-172 epitope induce distinct T-cell responses^24^. Although presented in similar conformations by both molecules, Tyr116 of HLA-B*57:03 reduces the space available to accommodate changes in KAFSPEVIPMF conformation on TCR ligation, requiring re-orientation of Tyr116, and impacting TCR selection through altered TCR-pHLA affinity^25^. In contrast, our structural analyses encompass peptides of optimal length to be contained within the antigen-binding cleft (9mers). These peptides occupy the cleft without marked bulging or overhang^25,45–48^, however polymorphic residues of the cleft cause distinct amino acid residue orientations. Our data suggest that 9-10mer HLA-B57/58 HIV-1 epitopes possessing Arg or Lys at PΩ-2 such as QATQDVKNW (Gag308-316), and its escape variants, and AVRHFPRIW (Vpr30-38)^21,23^ may adopt distinct conformations across the B57 family and generate structurally distinct targets for T-cell responses, which, in conjunction with quantitative differences in contribution to the immunopeptidome, may in turn explain the clinical differences in patients with these allotypes.

The observed alloreactivity between HLA-B*57:01 and HLA-B*58:01 further indicates that the differences in presentation of the self-proteome described are sufficient to alter recognition reminiscent of alloresponses between HLA-B*44:02 and HLA-B*44:03 molecules, which differ by a single residue buried within the antigen-binding cleft (Asp156Leu) and induce alloreactivity when mismatched in transplant scenarios^49,50^. Although the residue 156 polymorphism does not impact the primary anchor pockets of these allotypes, resulting in highly similar peptide-binding motifs and immunopeptidomes, differences are sufficient to stimulate alloresponses and are augmented by the ability of these allotypes to present identical peptides in structurally distinct conformations^28^. Thus, caution may be necessary when embarking on transplants between individuals bearing HLA-B57/58 mismatches.

In summary, we present the first comprehensive investigation of the impact of micropolymorphism on the immunopeptidome of HLA class I molecules. This has involved detailed analysis of ligand-binding specificity, qualitative and quantitative analysis of the immunopeptidomes of three clinically important HLA-B57 family members, and structural and functional characterisation of these differences. We show that micropolymorphism influences the immunopeptidome at several interlinked levels: (i) the repertoire of displayed peptides; (ii) quantity of displayed peptides; (iii) stability of pHLA, which will impact on the dynamics of the immunopeptidome; and (iv) conformation of pHLA. Importantly, such differences may amplify the responding T-cell repertoire against pathogens in heterozygous individuals but restrict transplantation options when considering micropolymorphic mismatches between donor-recipient pairings. Moreover, these findings suggest a need to look beyond qualitative analysis of the peptide repertoire when trying to unravel the nature of HLA-peptide presentation that dictates susceptibility to viral infection, autoimmunity, transplant rejection and drug hypersensitivity.

## Methods

### Ethics

Healthy individuals (*n* = 6) expressing either HLA-B*57:01, HLA-B*58:01 or both were recruited for the study. Ethics was granted from both Monash University (DHS numbers) and the Australian Bone Marrow Donor Registry (AP numbers) human ethics committees. Informed consent was obtained from all participants and research was performed in compliance with ethical regulations for the use of human samples.

### Peripheral blood mononuclear cell isolation

Peripheral blood samples were collected in heparinised vacutainer tubes and peripheral blood mononuclear cells (PBMCs) were isolated by Ficoll–Paque (GE Healthcare, Sweden) and density gradient centrifugation and cryopreserved until required.

### Cell lines and culture

C1R.B*57:01, C1R.B*57:03 and C1R.B*58:01 are B-LCLs, derived from the C1R cell line that expresses reduced amounts of HLA class I (reduced HLA-A2, reduced HLA-B35 and normal HLA-Cw4^51,52^), and transfected with HLA-B*57:01, HLA-B*57:03 or HLA-B*58:01 cDNA cloned into the pcDNA3.1(−) vector (Invitrogen, USA)^15^.

T-cell alloreactivity assays included the following B-LCLs (9053^53^: A*33:03, B*44:03 and C*14:03; T241: A*23:01, B*07:02, B*41:01, C*07:02 and C*08:02; A21: A2 and B40) and transfected cell lines (C1R.parental/A*01:01/A*02:01/A*03:01/B*07:02/B*08:01/B*15:01/B*44:02/B*44:03/B*57:01/B*58:01). C1R transfectants were produced within the McCluskey laboratory (Peter Doherty Institute, University of Melbourne, Victoria); T241 and A21 were provided by the Victorian Transplantation and Immunogenetics Service (West Melbourne, Victoria).

All cell lines were cultured in RF10 [RPMI 1640 (Life Technologies, USA) supplemented with 10% foetal calf serum (Sigma, St Louis, USA), 7.5 mM HEPES (MP Biomedicals, Germany), 100 U mL^−1^ Pen-Strep (benzyl-penicillin/streptomycin, Life Technologies, USA), 2 mM l-glutamine (MP Biomedicals, Germany), 76 μM β-mercaptoethanolamine (Sigma-Aldrich, USA) and 150 μM non-essential amino acids (Life Technologies, USA)] at 37 °C, 5% CO_2_. Maintenance of transfected HLA expression during long-term culture was facilitated by addition of Geneticin, 0.4–0.5 mg mL^−1^ (G418; Life Technologies, USA), or Hygromycin B, 0.2–0.3 mg mL^−1^ (Life Technologies, USA). Increased HLA class I expression (as compared to C1R parental) was confirmed via flow cytometry after staining with the HLA class I pan-specific monoclonal antibody W6/32^54^ (produced in house from the W6/32 hybridoma) and Goat F(ab′)2 Anti-Mouse IgG(H + L), Human ads-PE (1:500, catalogue number 1032-09, Southern Biotech, USA). All cell lines were tested for mycoplasma contamination.

### Motif characterisation and data set comparisons

We had previously isolated and sequenced peptide ligands from HLA class I of 10^9^ C1R-B*57:01, C1R-B*57:03 and C1R-B*58:01 cells by LC-MS/MS using an information-dependent acquisition (IDA) strategy^15^. Spectra were assigned with ProteinPilot^TM^ software version 5.0 (SCIEX, USA) searching against the reviewed Swiss-Prot human proteome (accessed November 2017) and peptide identities determined subject to strict bioinformatic criteria, assigning confidence values to each peptide and including the use of a decoy database to calculate the FDR. Peptides known to bind the endogenous HLA class I of C1R cells (HLA-C*04:01 and HLA-B*35:03)^55^ were removed before subsequent analysis. A further list of peptide contaminants, generated by comparison of a large number of similar elution experiments for MHC I and MHCII were also disregarded, in addition to peptides of the HLA proteins (Supplementary Data 3). To characterise the peptide-binding motif of each HLA allotype, distinct peptides identified within three biological replicate experiments were filtered using a confidence cut-off for a 5% local FDR (95.2–97) and pooled to generate a single data set for analysis. The frequency of peptides (non-redundant by sequence) of specific lengths and/or possessing a particular amino acid at a specified position within the peptide was then calculated and sequence motifs generated for 9–11 residue peptides. Heat maps of the inter-position coupling matrices were generated for each of the 9mer, 10mer and 11mer peptides. Statistical coupling of two sites in the peptide was defined as the degree to which amino acid frequencies at one site change in response to a perturbation of frequencies at a second site^56^. Coupling matrices were processed and analysed with custom Perl and MATLAB (The MathWorks Inc., Natick, MA) scripts^57^. Scripts are available (https://github.com/jlmendozabio/covariation_stats).

For PCA based on amino acid physicochemical properties, 4 quantitative biophysical properties (molecular weight, hydropathy index, surface area and isoelectric point) were determined for each position of the peptide. We also incorporated 20 additional parameters at each amino acid position describing the identity of the amino acid present. From the points generated by the PCA, a two-dimensional kernel density plot was used to more clearly display large numbers of peptides. These variables were processed for peptides of 9–11 amino acids in length from each HLA allotype. For each different combination of PC scores, we performed clustering by *k*-means clustering. Silhouette analysis was used to provide a quantitative assessment of cluster similarity. On the basis of peaks in silhouette coefficient across the number of clusters, peptides were assigned into one of the two distinctive clusters present in all allotypes and for all peptide lengths by using *k*-means clustering (*k* = 2) on the first two principal components. To visualise the sequence motifs present in each cluster, peptide sequences were extracted from each cluster and their motifs generated based on residue frequency as described above. These analyses were performed using a custom R script^58–61^ (available at https://github.com/ParhamLab/PeptidePCA/tree/master/R).

Amino acid enrichment/regulation over prevalence in the human proteome was determined using the icelogo v1.2 stand-alone software via the static reference method (reference *Homo sapiens* Swiss-Prot means), and is depicted as FC (FC = prevalence in data set/prevalence in human proteome) for enriched amino acids, and converted FC (FC_con_ = −1/FC) for negatively regulated residues^31^. FC or FC_con_ was only depicted where the *Z*-score fell outside the confidence interval for a *p*-value of 0.05. Post-translational modifications of peptides were not considered during motif analysis.

To perform sequence-based comparison of data sets for overlap within the peptide repertoire, all peptides in a data set identified with a confidence ≥ 95 were included. Peptides identified with a confidence > 20 were also included if, and only if, they appeared in a compared data set with a confidence ≥ 95. Modifications were considered in overlap analysis.

### Purification of HLA-peptide complexes

C1R transfectants were grown to high density in 100 mL RF10 containing 0.5 mg mL^−1^ G418 in T175 tissue culture flasks (Greiner Bio-One International AG, Austria). Cells were harvested in batches of 10^8^ cells by centrifugation (1200 × *g*, 20 min, 4 °C), washed twice in chilled phosphate-buffered saline and frozen on dry ice for 15 min or by submersion in liquid nitrogen. Pelleted cells were stored at −80 °C until time of use. Detergent-based lysis was performed by resuspending cell pellets in 5 mL lysis buffer [0.5% IGEPAL (Sigma-Aldrich, USA), 50 mM Tris, pH 8, 150 mM NaCl (Merck-Millipore, Germany) and protease inhibitors (Complete Protease Inhibitor Cocktail Tablet [1 tablet per 50 mL solution]; Roche Molecular Biochemicals, Switzerland)] and incubating for 45 min at 4 °C with slow end-over-end mixing. Lysates were cleared by centrifugation at 16 000 × *g* for 20 min at 4 °C.

HLA-peptide complexes were immunoaffinity purified from cell lysates using 1 mg W6/32 monoclonal antibody crosslinked to protein A sepharose^6^. Bound complexes were eluted with 2 mL 10% acetic acid. The eluted mixture of peptides, class I heavy chain and β_2_-microglobulin (β_2_m) was fractionated on a 4.6 mm internal diameter × 50 mm long monolithic reversed-phase (RP) C_18_ high-performance liquid chromatography (HPLC) column (Chromolith Speed Rod, Merck-Millipore, Germany) utilising an ÄKTAmicro™ HPLC system (GE Healthcare, UK; Unicorn v5.11 software) and using a mobile phase consisting of buffer A (0.1% trifluoroacetic acid (TFA) [Thermo Scientific, USA]) and buffer B (80% acetonitrile (ACN) [Fisher Scientific, USA] and 0.1 % TFA), running at 1 mL min^−1^ with a gradient of B of 2–40% over 4 min, 40–45% over 4 min and 45–99% over 2 min, collecting 500 μL fractions. Three fraction pools were generated and vacuum concentrated for MS analysis. Ultraviolet absorbance of eluted material was monitored at 215 nm. The relative amount of HLA purified was measured as the area under the curve for the β_2_m.

### MRM quantification of HLA-bound peptides

Fraction pools from the RP-HPLC purification were concentrated using a speed vacuum concentration system (LABCONCO, USA). MRM detection was performed using an AB SCIEX QTRAP 5500 mass spectrometer, equipped with a Tempo nanoLC (Eksigent) autosampler and cHiPLC nanoflex (Eksigent) and utilising Analyst 1.6 (SCIEX) software. Samples were injected and loaded onto a trap column (200 µm × 0.5 mm ChromXP C_18_-CL packed with 3 µm particles, nominal pore size 120 Å) at a flow rate of 5 µL min^−1^ in 98% buffer A (0.1% formic acid in water), 2% buffer B (95% ACN and 0.1% formic acid in water) for 10 min. Samples were eluted from the trap column and over a cHiPLC column (75 µm × 15 cm ChromXP C_18_- packed with 3 µm particles, nominal pore size 120 Å) at 300 nL min^−1^ using the following gradient conditions: 0–3 min 2–10% B, 3–62 min 10–50% B, 62–65 min 40–80% B, 65–70 min hold at 80% B, 70–73 min 80–2% B, followed by equilibration at 2% B for 7 min. The QTRAP 5500 was operated in MRM mode in unit resolution for Q1 and Q3, coupled to an IDA criterion set to trigger an EPI scan (10 000 Da s^−1^; rolling CE; unit resolution) following any MRM transition exceeding 600 counts. Triggering MRM transitions were ignored for the subsequent 6 s.

The detection of all three to four transitions overlapping at a particular retention time, accompanied by MRM triggered-MS/MS fragmentation in at least one experiment, was used as an indicator of peptide presence. Fragment ion intensity rankings were compared to those in initial IDA-based discovery experiments using a spectral library generated from data for the three HLA allotypes using Skyline 64 bit 3.5.0.9319 (MacCoss Laboratory^62^) and calculated as a dot product value. Peptides detected in a sample without MS/MS validation were considered valid if the retention time (RT) was ±1.5 min of the average RT for MS/MS validated appearances of that peptide and the dot product value was >0.7. Relative peptide abundance was calculated as the total area under the curve for the detected transitions using Skyline software, normalised to the amount of purified HLA from which the sample was derived, allowing comparison between samples in the absence of absolute quantitation.

### Allogeneic T-cell stimulation

T-cell cultures were generated from 5 × 10^6^ responder PBMCs stimulated with 2.5 × 10^6^ irradiated allogeneic PBMCs. Culture medium was supplemented with 20 U mL^−1^ recombinant human IL-2 (Cetus) and changed every 2–3 days to maintain saturating levels of nutrients and growth factors. On day 13, 2 × 10^5^ responders from the T-cell culture were restimulated with 10^5^ B-LCLs expressing allo-HLA. After 2 h of coincubation (37 °C, 5% CO_2_), 10 µg mL^−1^ Brefeldin A (Sigma-Aldrich, USA) was added for a further 4 h. Responder CD8^+^ T cells were stained with anti-CD8 PerCP-Cy5.5 (1:20, clone SK1, catalogue number 341051, Becton Dickinson [BD] Biosciences, USA), anti-CD4 PE (1:20, clone RPA-T4, catalogue number 555347, BD Biosciences) and a viability dye (1:750, LIVE/DEAD™ Fixable Aqua Dead Cell Stain, 405 nm excitation, catalogue number L34957, Thermo Fisher), fixed with 1% paraformaldehyde (ProSciTech, Australia) and permeabilised with 0.3% Saponin (Sigma-Aldrich, USA) containing anti-IFNγ PE-Cy7 (1:250, clone B27, catalogue number 557643, BD Biosciences, USA) and acquired on a LSRII flow cytometer (BD, USA) utilising BD FACSDIVA™ software. The percentage of allo-specific CD8^+^ T cells producing IFNγ was analysed using FlowJo software (Tree Star Inc., USA)^36^, utilising the gating strategy shown in Supplementary Fig. 10. Sample numbers were dictated by availability of HLA-B*57:01/HLA-B*58:01 PBMC.

### Recombinant HLA-peptide complex generation

The HLA-B*57:01, HLA-B*57:03, HLA-B*58:01 and *β*_*2*_*m* genes were sub-cloned into the pET-30 expression vector and were expressed into inclusion bodies separately in *Escherichia coli*. The HLA complexes were refolded in the presence of the peptides listed in Table 1 and purified as described previously^63^. Briefly, 90 mg HLA heavy chain was refolded by rapid dilution in a solution containing 3 M urea (Sigma-Aldrich, USA), 100 mM Tris-HCl, pH 8.0 (Sigma-Aldrich, USA), 400 mM l-arginine-HCl, 5 mM reduced glutathione (Sigma-Aldrich, USA) and 0.5 mM oxidised glutathione (Sigma-Aldrich, USA) in the presence of 30 mg β_2_m and 10 mg of the appropriate peptide for 48 h. The refolded HLA-peptide complexes were dialysed into 10 mM Tris, pH 8.0, and purified by size-exclusion chromatography using HiLoad 16/60 Superdex 200 pg (GE Healthcare, USA) columns on an AKTA Purifier (GE Healthcare, USA) FPLC chromatography systems in 10 mM Tris, pH 8.0, and 150 mM NaCl buffer. Final purification was by anion exchange using a HiTrap Q Fast Flow column (GE Healthcare, USA) on the same AKTA system in 10 mM Tris pH 8.0 buffer with a NaCl gradient from 0 to 500 mM over 45 min.

### Thermal melt experiments

Thermal stability assays were performed at 0.5 and 1 mg mL^−1^ HLA-peptide complex in 10 mM Tris and 150 mM NaCl, pH 8.0 in a reaction volume of 25 μL in duplicate except where otherwise indicated. Protein unfolding was monitored by the addition of the fluorescent dye SYPRO^®^ Orange (Sigma-Aldrich, USA) at 10× concentration. Refolded complexes were heated from 35 to 90 °C at a heating rate of 1 °C min^−1^ in the Real Time Detection system (Rotor-Gene^®^ Q, QIAGEN) and fluorescence intensity was measured using an excitation wavelength of 530 nm and emission at 555 nm.

### X-ray crystallography

The peptide sequences crystallised in complex with HLA-B*57:01, HLA-B*57:03 and HLA-B*58:01 are noted in Table 1. HLA-peptide complexes were concentrated to ~10 mg mL^−1^ and crystallised at 294 K by the hanging-drop vapour-diffusion method from a solution comprising 12–20% PEG 4000, 0.2 M ammonium acetate and 0.1 M tri-sodium citrate pH 5.4–5.6. Prior to data collection, crystals were equilibrated in reservoir solution with 10% glycerol added as a cryoprotectant and then flash-cooled in a stream of liquid nitrogen at 100 K. Data sets were collected at the MX2 beamline (Australian Synchrotron, Victoria). The data were recorded on a Quantum-315 CCD detector and were integrated and scaled using MOSFLM and SCALA from the CCP4 programme suite^64–66^. Details of the data processing statistics are summarised in Table 2. Phases for the structures were determined by molecular replacement as implemented in PHASER^67^ with HLA-B*57:01-LF9 used as the search model (Protein Data Bank accession number: 2RFX^34^). Refinement of the models proceeded with iterative rounds of manual building in COOT^68^, refinement in PHENIX^69^ and validation with MOLPROBITY^69^. Refinement statistics are summarised in Table 2.

### Code availability

Scripts for co-variation analysis and PCA are available at https://github.com/jlmendozabio/covariation_stats and https://github.com/ParhamLab/PeptidePCA/tree/master/R.

## Electronic supplementary material

Supplementary Information

Description of Additional Supplementary Files

Supplementary Data 1

Supplementary Data 2

Supplementary Data 3

Reporting Summary

## Data Availability

Proteomics data sets analysed during this study have been deposited to the ProteomeXchange Consortium via the PRIDE^70^ partner repository with the data set identifiers PXD008570 (C1R.B*57:01 LC-MS/MS), PXD008571 (C1R.B*57:03 LC-MS/MS), PXD008572 (C1R-B*58:01 LC-MS/MS) and PXD009850 (LC-MRM). Coordinates and structure factors were deposited in the PDB with the following codes: B5701-LSSPVTKSW 5VUD; B5701-LTVQVARVW 5VUE; B5701-LTVQVARVY 5VUF; B5703-LSSPVTKSW 5VVP; B5703-LTVQVARVW 5VWD; B5703-LTVQVARVY 5VWF; B5801-LSSPVTKSW 5VWH; and B5801-LTVQVARVW 5VWJ. All other data are available from the corresponding author on reasonable request.
