# Supplementary material for: HLA-B57 micropolymorphism defines the sequence and conformational breadth of the immunopeptidome
Source: Nat Commun. 2018 Nov 8;9:4693. doi: 10.1038/s41467-018-07109-w (PMC6224591; doi:10.1038/s41467-018-07109-w)
Supplement: Supplementary file 1 — Supplementary Information [file 41467_2018_7109_MOESM1_ESM.pdf]

HLA-B57 micropolymorphism defines the sequence and conformational breadth of the immunopeptidome

Illing *et al.*

Supplementary Information

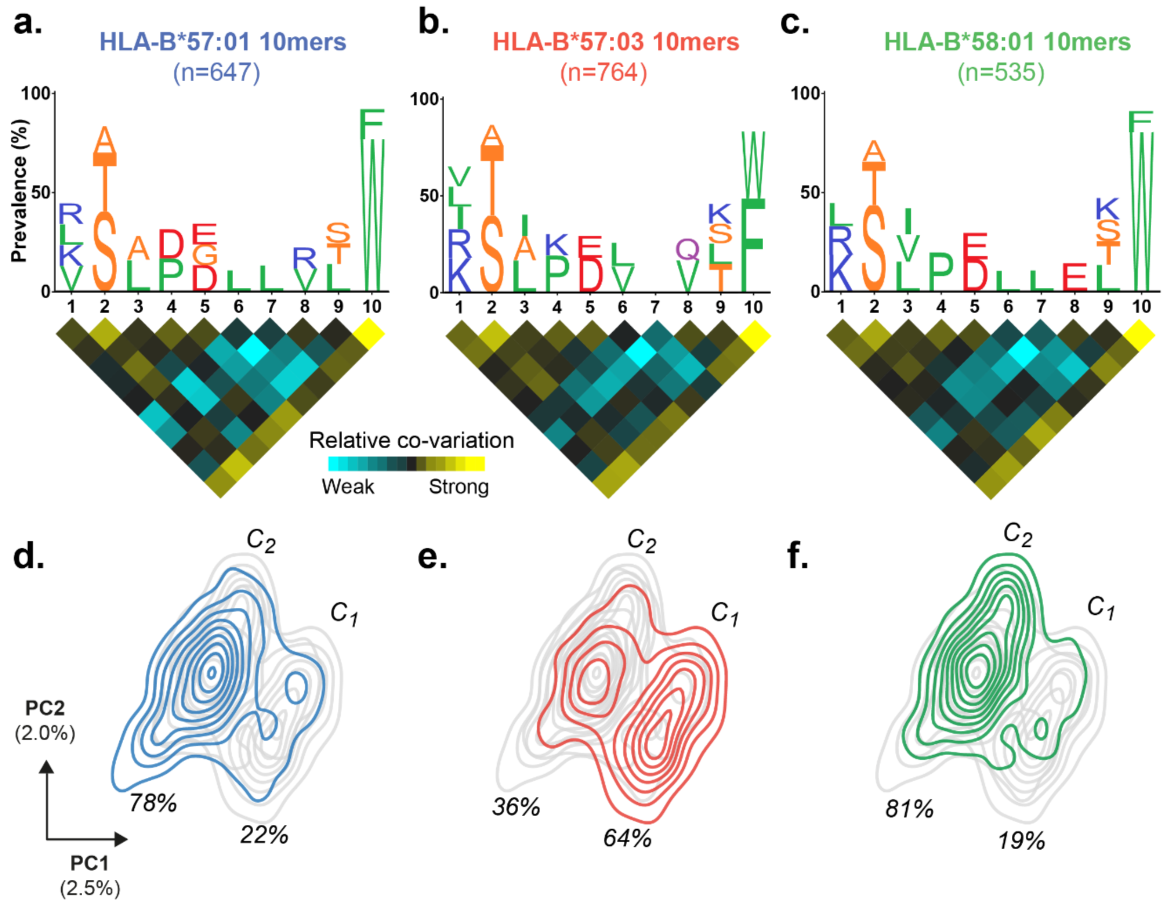

Supplementary Figure 1: HLA-B*57:01, HLA-B*57:03 and HLA-B*58:01 sample 10mer peptides with similar physicochemical properties, but with different biases at the C-terminus.

(a-c) Sequence motifs of 10mer peptides for each allomorph with accompanying co-variation heat maps. Amino acids are represented by the single letter code, letter height is scaled to prevalence and colours represent small (orange), hydrophobic (green), polar (magenta), negatively charged (red) and positively charged (blue) residues. Only amino acids present with 10 % or greater prevalence are depicted. n is the number of 10mer peptides within the data set. For the heat maps, square colour reflects the relative co-variation at, or between, each position in the peptide (aqua indicates weak co-variation and yellow indicates strong co-variation). (d-f) Principal component analysis density plots showing the distribution of 10mer peptides across PC1 and PC2 for HLA-B*57:01 (d), HLA-B*57:03 (e) and HLA-B*58:01 (f). Coloured lines represent the named allomorph (HLA-B*57:01 – blue, HLA-B*57:03 – red, HLA-B*58:01 – green) overlaid on the distribution of the remaining two allomorphs (grey lines). Two major clusters (c_1 and c_2) were defined using k-means clustering (k = 2), and the designation of the clusters as c_1 and c_2 is matched to the PCA of 9mer peptides based on similarity of feature contributions. Variation of PC1 and PC2 are shown in parentheses.

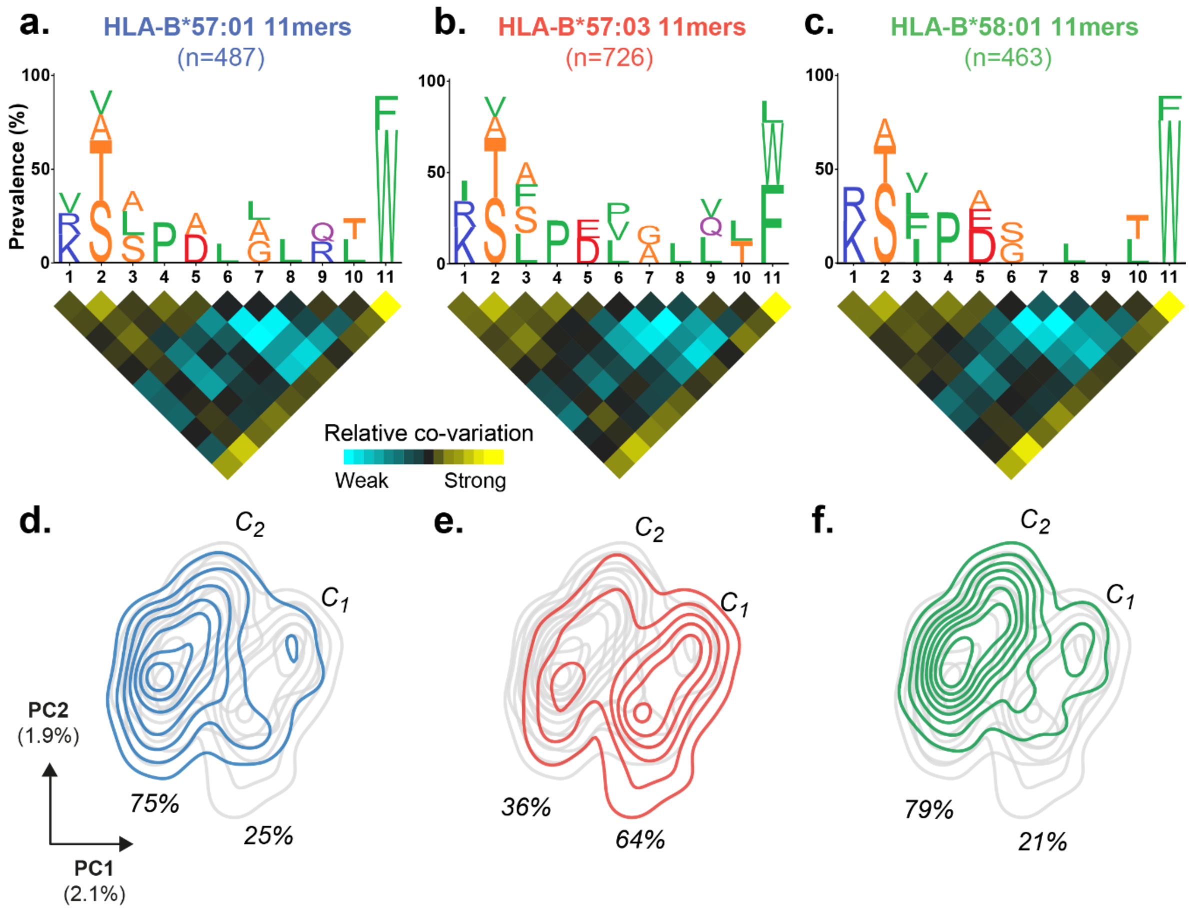

Supplementary Figure 2: HLA-B*57:01, HLA-B*57:03 and HLA-B*58:01 sample 11mer peptides with similar physicochemical properties, but with different biases at the C-terminus.

(a-c) Sequence motifs of 11mer peptides for each allomorph with accompanying co-variation heat maps, highlighting pairs of positions that are coupled. Amino acids are represented by the single letter code, letter height is scaled to prevalence and colours represent the following properties; small (orange), hydrophobic (green), polar (magenta), negatively charged (red), positively charged (blue). Only amino acids present with 10 % or greater prevalence are depicted. n is the number of 11mer peptides within the data set. For the heat maps, square colour reflects the relative co-variation at, or between, each position in the peptide (aqua indicates weak co-variation and yellow indicates strong co-variation). (d-f) Principal component analysis density plots showing the distribution of 11mer peptides across PC1 and PC2 for HLA-B*57:01 (d), HLA-B*57:03 (e) and HLA-B*58:01 (f). Coloured lines represent the named allomorph (HLA-B*57:01 – blue, HLA-B*57:03 – red, HLA-B*58:01 – green) overlaid on the distribution of the remaining two allomorphs (grey lines). Two major clusters (c_1 and c_2) were defined using k-means clustering (k = 2), and the designation of the clusters as c_1 and c_2 is matched to the PCA of 9mer peptides based on similarity of feature contributions. Variation of PC1 and PC2 are shown in parentheses.

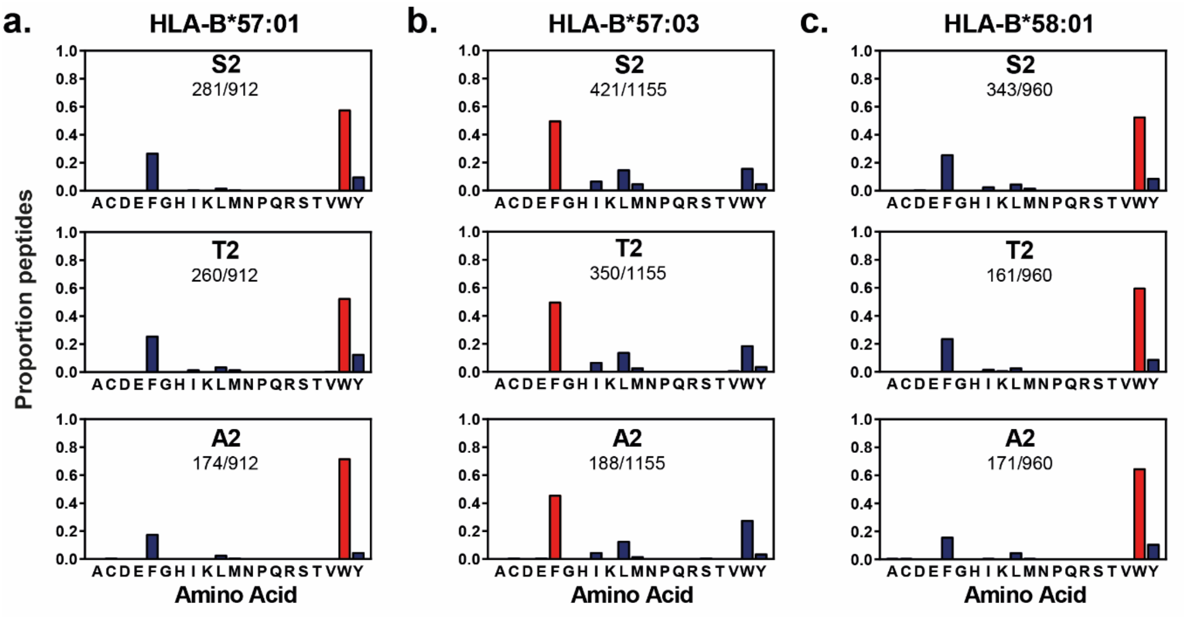

Supplementary Figure 3: Covariation analysis.

The distribution of amino acids at P9 in 9mer peptides with serine (top row), threonine (centre row) or alanine (bottom row) at P2. Results are shown for peptides bound by HLA-B*57:01 (a), HLA-B*57:03 (b) and HLA-B*58:01 (c). The most abundant residue at P9 for each subset is highlighted in red.

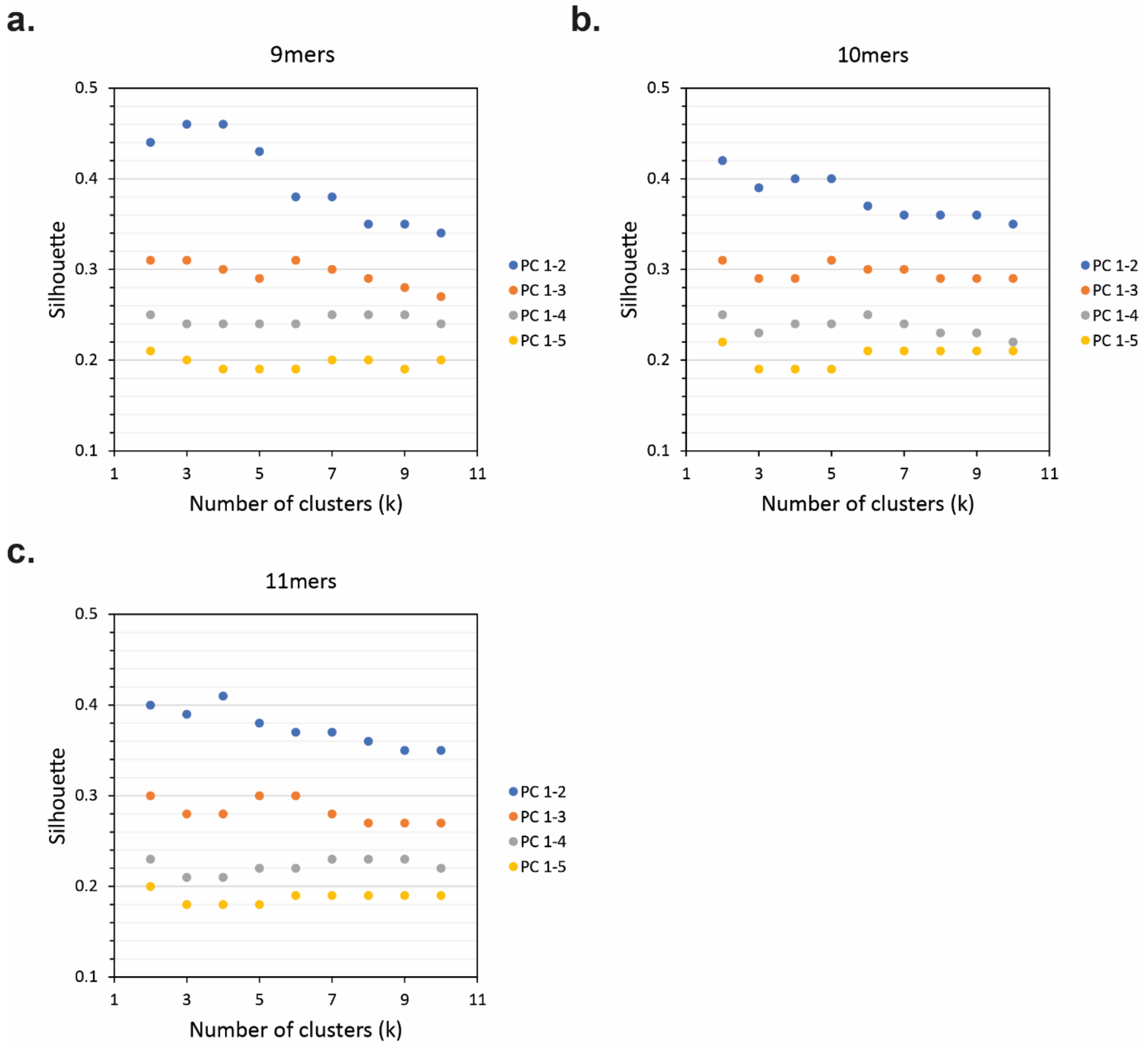

Supplementary Figure 4: Silhouette analysis for different k in k-means clustering.

Silhouette analysis of clusters determined by k-means clustering utilising PC1-2, PC1-3, PC1-4 and PC1-5 with k values of 2-10. When using PC1-2 alone, optimal segregation of clusters was observed at k values of 2-5, beyond which silhouette width reduced. For 10mers greatest silhouette width was achieved at k=2. For 9mers and 11mers this was slightly improved at k=4, showing that whilst these data can all be described by 2 clusters, substructure that may become more evident with greater data set size also exists. Use of further PCs reduced the silhouette score.

a.

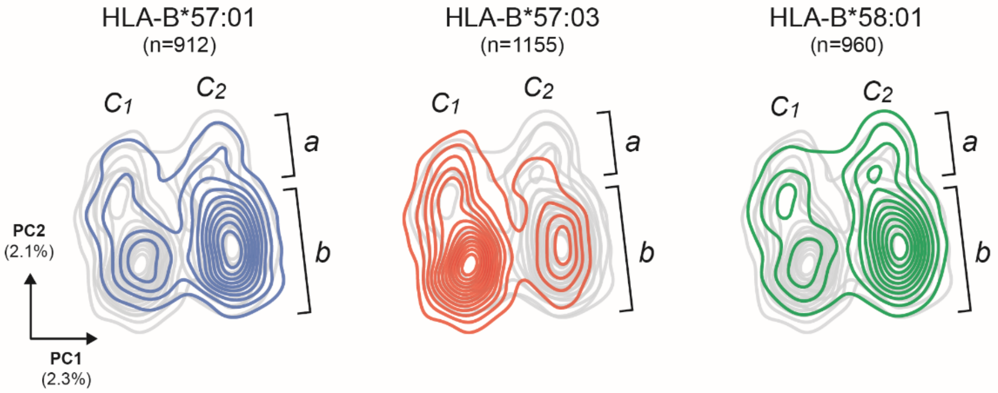

b.

9mers	HLA-B*57:01 (n=912)			HLA-B*57:03 (n=1155)			HLA-B*58:01 (n=960)		
	a	*b*	Total	*a*	*b*	Total	*a*	*b*	Total
Cluster 1	13	19	32	26	50	76	16	17	33
Cluster 2	19	49	68	7	17	24	23	44	67
Total	32	68	100	33	67	100	39	61	100

c.

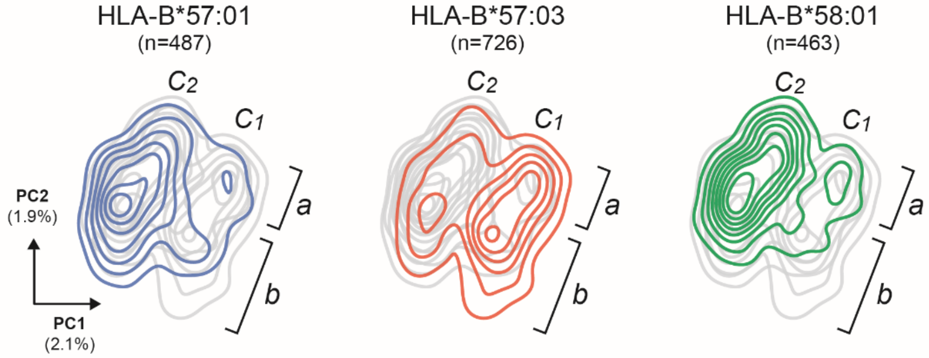

d.

11mers	HLA-B*57:01 (n=487)			HLA-B*57:03 (n=726)			HLA-B*58:01 (n=463)		
	a	*b*	Total	*a*	*b*	Total	*a*	*b*	Total
Cluster 1	12	13	25	30	33	63	12	8	20
Cluster 2	33	42	75	18	19	37	46	34	80
Total	45	55	100	48	52	100	58	42	100

Supplementary Figure 5: Sub-cluster position in PCA density plots for 9 and 11mer peptides.

PCA density plots showing the locations of sub-clusters when k=4 in k-means clustering for 9mer (a) and 11mer (c) peptides. Coloured lines represent the named allomorph (HLA-B*57:01 – blue, HLA-B*57:03 – red, HLA-B*58:01 – green) overlaid on the distribution of the remaining two allomorphs (grey lines). Proportions of peptides within each sub-cluster for 9mer and 11mer are shown in (b) and (d).

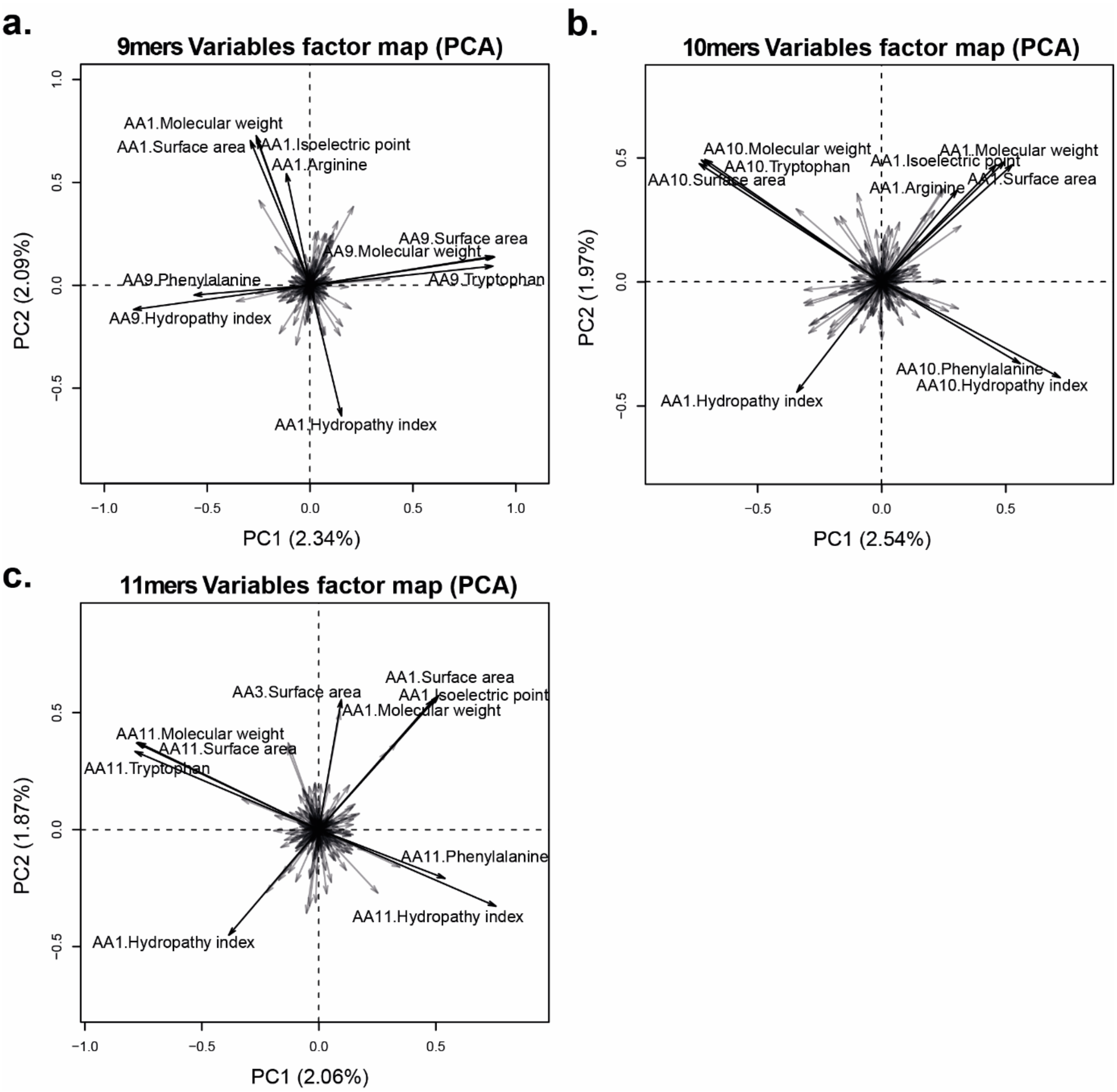

Supplementary Figure 6: PΩ, then P1, are the most influential positions in both the PCA and clustering.

A plot of feature contributions to PC1 and PC2 shown as vectors for PCAs for 9mer (a), 10mer (b) and 11mer (c) peptides. The top 10 strongest contributing features are in bold and labeled. PΩ properties here establish the axis in which the PCA plots forms two clusters, and clusters were nominated as c_1 and c_2 across the three independent PCA based on similarity in feature contribution (Fig. 1, Supplementary Fig. 1 and 2).

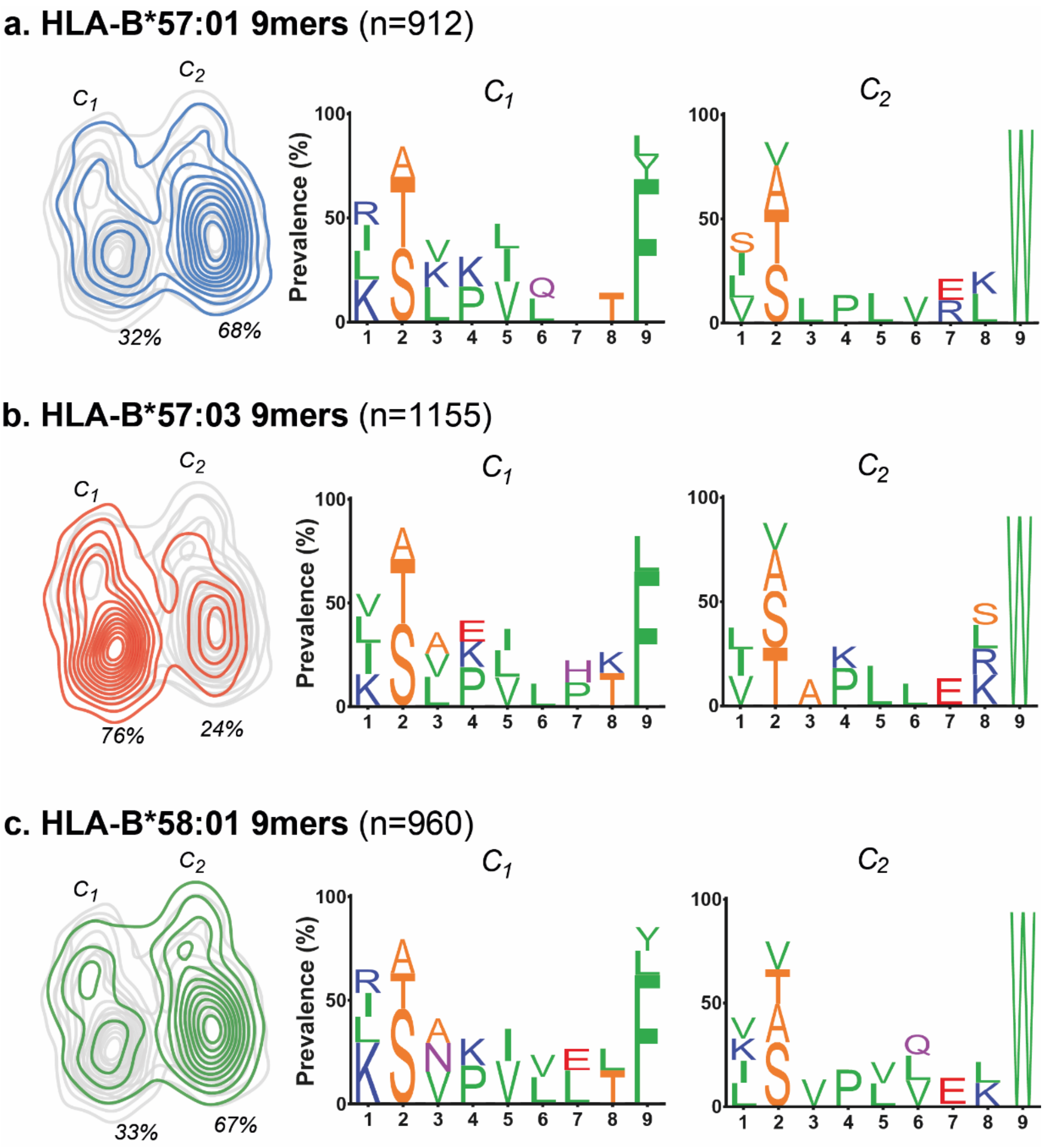

Supplementary Figure 7: PCA segregates HLA-B*57:01, HLA-B*57:03 and HLA-B*58:01 peptides into two clusters distinguished primarily by PΩ.

Sequence logos were generated for the 9mer peptides mapping to clusters c_1 and c_2 for HLA-B*57:01 (a), HLA-B*57:03 (b) and HLA-B*58:01 (c) as defined by k-means clustering (k = 2) (PCA plots are those shown in Fig. 1, coloured lines represent the named allomorph [HLA-B*57:01 – blue, HLA-B*57:03 – red, HLA-B*58:01 – green] overlaid on the distribution of the remaining two allomorphs [grey lines]). Amino acids are represented by the standard single letter code, letter height is scaled to amino acid prevalence, and colours represent the following properties; small (orange), hydrophobic (green), polar (magenta), negatively charged (red), positively charged (blue). Only amino acids present with 10 % or greater prevalence are depicted.

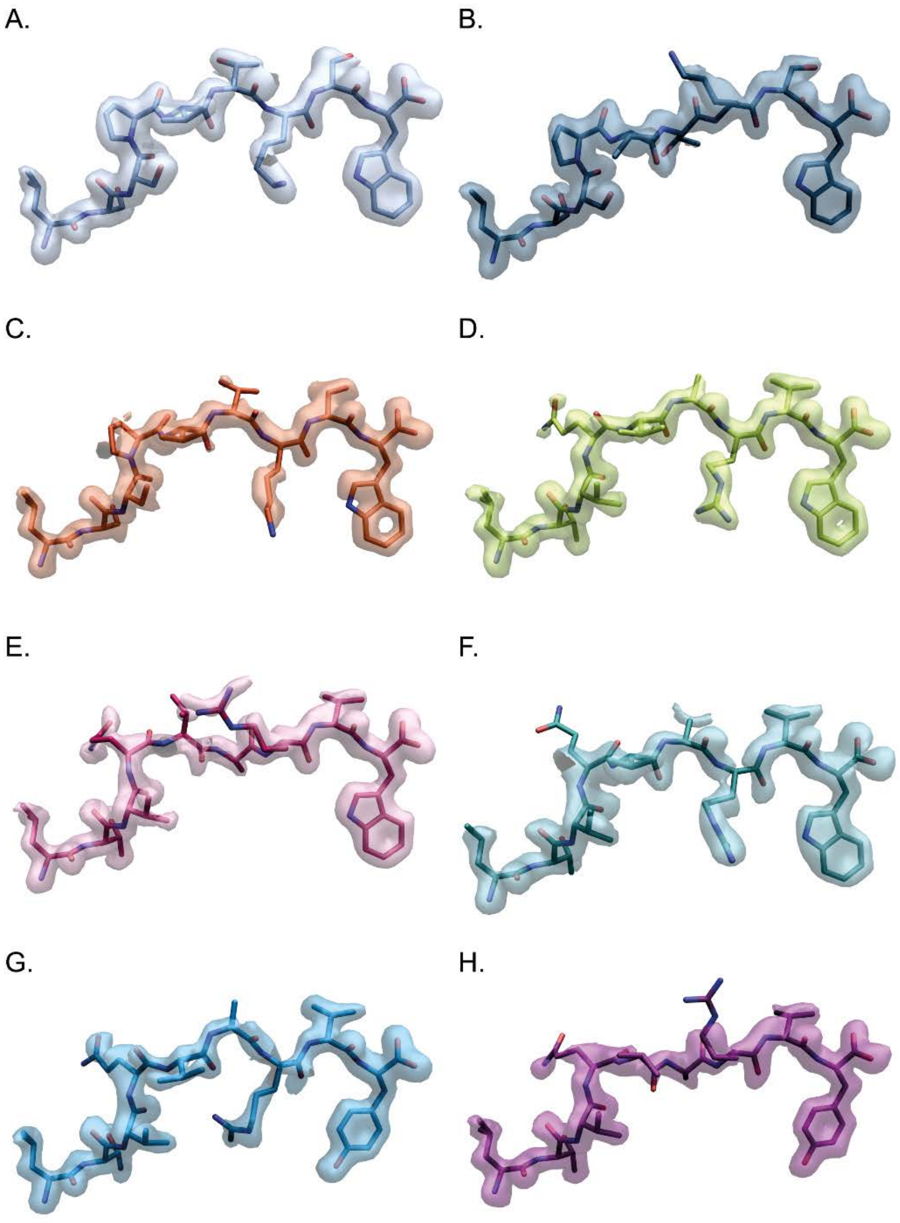

Supplementary Figure 8: Unbiased electron density of peptide structures solved.

F_o-F_c a_{calc} omit maps showing unbiased electron density for the respective peptides in the following structures at 3σ (1.5Å carve). A. HLA B*57:01-LSSPVTKSW B. HLA B*57:03-LSSPVTKSW C. HLA B*58:01-LSSPVTKSW D. HLA B*57:01-LTVQVARVW E. HLA B*57:03-LTVQVARVW F. HLA B*58:01-LTVQVARVW G. HLA B*57:01-LTVQVARVY and H. HLA B*57:03-LTVQVARVY.

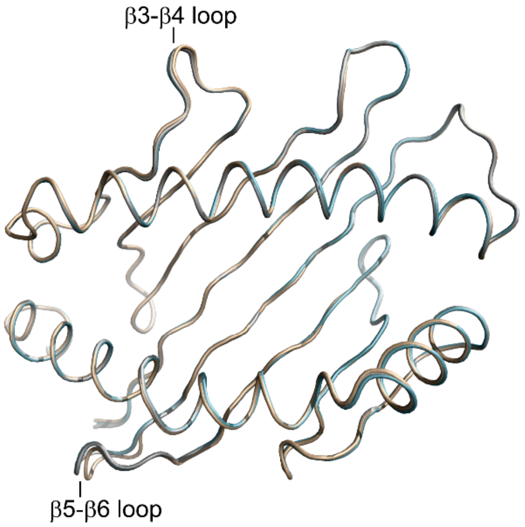

Supplementary Figure 9: Superposed Cα traces of the peptide binding grooves.

Superposed backbone traces of the structures of HLA-B*57:01 (grey), HLA-B*57:03 (cyan) and HLA-B*58:01 (orange) presenting the LSSPVTKSW peptide. The deviation at the β3-β4 and β5-β6 loops engendered by the Ala46Glu and Val103Leu substitutions in HLA-B*58:01 are labelled.

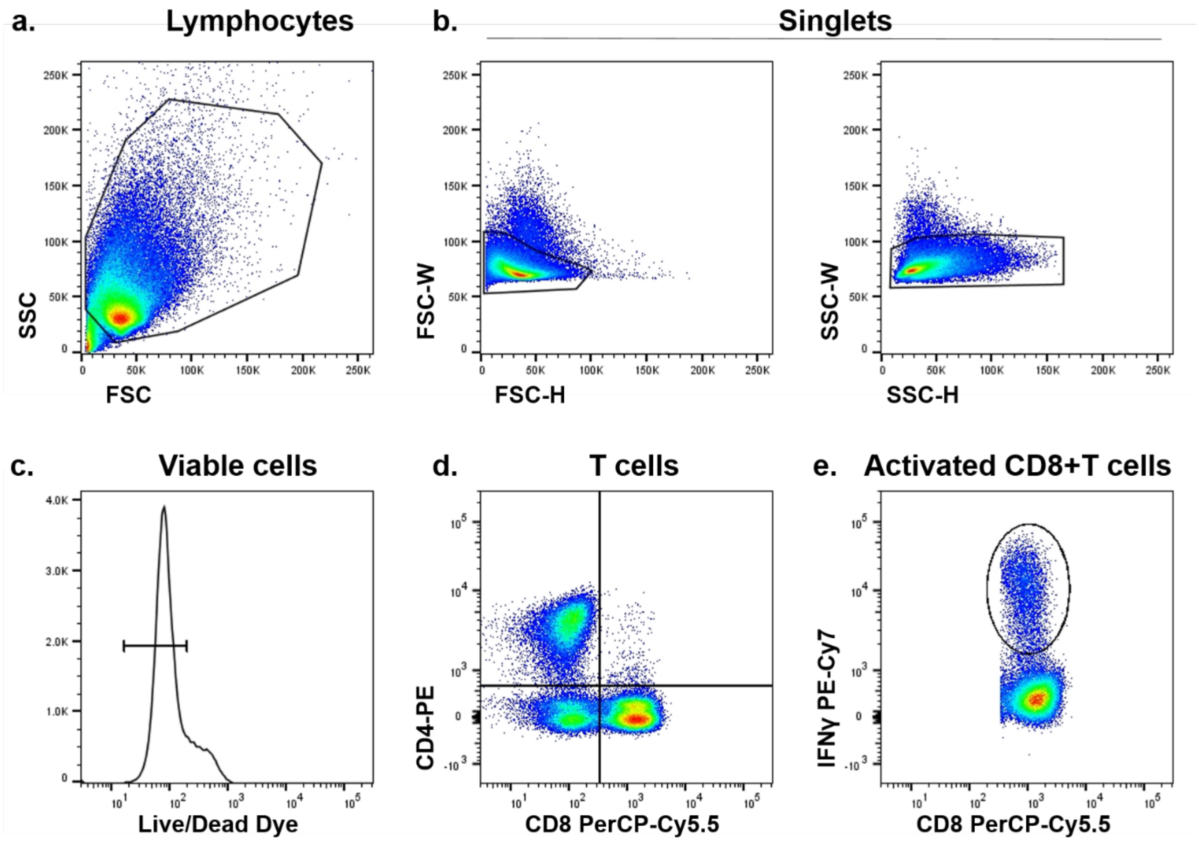

Supplementary Figure 10: Mixed lymphocyte reaction gating strategy.

Lymphocytes were gated on forward scatter (FSC) and side scatter (SSC) (a), and single cells gated using FSC-width (FSC-W) compared to FSC-height (FSC-H) then SSC-W and SSC-H (b). Viable cells isolated as those negative for staining with LIVE/DEAD™ Fixable Aqua Dead Cell Stain (c). Live T cells were phenotyped based on CD4 and CD8 expression (d) and the CD8+, CD4- quadrant selected for analysis of IFNγ expression (e).

Supplementary Table 1: Percentage of 9-11mer peptide ligands terminating in Trp and Phe residues

	9mers		10mers		11mers	
**Molecule**	**Trp**	**Phe**	**Trp**	**Phe**	**Trp**	**Phe**
**HLA-B*57:01**	62.4	22.1	76.7	15.0	70.0	18.1
**HLA-B*57:03**	21.6	49.5	34.4	47.8	33.6	43.1
**HLA-B*58:01**	62.1	20.8	80.2	10.7	75.4	13.4

Supplementary Table 2: Percentage of 9-11mer peptide ligands possessing Glu, Lys or Arg at PΩ-2

	9mers			10mers			11mers		
**Molecule**	**Glu**	**Lys**	**Arg**	**Glu**	**Lys**	**Arg**	**Glu**	**Lys**	**Arg**
**HLA-B*57:01**	9.65	6.14	9.76	7.88	7.57	11.1	5.34	4.93	11.3
**HLA-B*57:03**	9.96	2.25	1.21	8.64	1.83	1.57	4.41	1.38	2.07
**HLA-B*58:01**	11.7	2.60	1.15	13.8	1.68	2.24	8.21	1.94	3.67

Supplementary Table 3: MLR matrix

	Responder			Stimulator				
**MLR No.**		**[a]HLA-A or -A***	**[a]HLA-B or -B***		**[a]HLA-A or -A***	**[a]HLA-B or -B***	**APCs expressing matched/mismatched alleles**	**APCs used for background subtraction[#]**
1	DHS009	01:01	08:01, 57:01	AP013	02:01, 33:03	15:01, 58:01	C1R.A*02:01, C1R.B*15:01, C1R.B*58:01; 9053 B-LCL (A*33:03, *B*44:03*)	C1R.parental; C1R.B*44:03
2				AP015	03:01	40:06, 58:01	C1R.A*03:01, C1R.B*58:01; A21 B-LCL (*A2*, B40)	C1R.parental; C1R.A*02:01
3	DHS011	1, 23	44, 57	AP013	02:01, 33:03	15:01, 58:01	C1R.A*02:01, C1R.B*15:01, C1R.B*58:01; 9053 B-LCL (A*33:03, *B*44:03*)	C1R.parental; C1R.B*44:03
4				AP015	03:01	40:06, 58:01	C1R.A*03:01, C1R.B*58:01; A21 B-LCL (*A2*, B40)	C1R.parental; C1R.A*02:01
5	AP013	02:01, 33:03	15:01, 58:01	DHS009	01:01	08:01, 57:01	C1R.A*01:01, C1R.B*08:01, C1R.B*57:01	C1R.parental
6				DHS011	1, 23	44, 57	C1R.A*01:01, C1R.B*44:02, C1R.B*44:03, C1R.B*57:01; T241 B-LCL (A*23:01, *B*07:02*, B*41:01)	C1R.parental; C1R.B*07:02
7	AP015	03:01	40:06, 58:01	DHS009	01:01	08:01, 57:01	C1R.A*01:01, C1R.B*08:01, C1R.B*57:01	C1R.parental
8				DHS011	1, 23	44, 57	C1R.A*01:01, C1R.B*44:02, C1R.B*44:03, C1R.B*57:01; T241 B-LCL (A*23:01, *B*07:02*, B*41:01)	C1R.parental; C1R.B*07:02
9	DHS006	02:01, 29:02	57:01	DHS009	01:01	08:01, 57:01	C1R.A*01:01, C1R.B*08:01, C1R.B*57:01	C1R.parental
10				DHS011	1, 23	44, 57	C1R.A*01:01, C1R.B*44:02, C1R.B*44:03, C1R.B*57:01; T241 B-LCL (A*23:01, *B*07:02*, B*41:01)	C1R.parental; C1R.B*07:02
11				AP013	02:01, 33:03	15:01, 58:01	C1R.A*02:01, C1R.B*15:01, C1R.B*58:01; 9053 B-LCL (A*33:03, *B*44:03*)	C1R.parental; C1R.B*44:03

12				AP015	03:01	40:06,<br>58:01	C1R.A*03:01, C1R.B*58:01;<br>A21 B-LCL (_A2_, B40)	C1R.parental;<br>C1R.A*02:01
13	AP012	25:01,<br>26	57:01,<br>58:01	DHS009	01:01	08:01,<br>57:01	C1R.A*01:01, C1R.B*08:01, C1R.B*57:01	C1R.parental
14				DHS011	1, 23	44, 57	C1R.A*01:01, C1R.B*44:02, C1R.B*44:03,<br>C1R.B*57:01;<br>T241 B-LCL (A*23:01, _B*07:02_, B*41:01)	C1R.parental;<br><br>C1R.B*07:02
15				AP013	02:01,<br>33:03	15:01,<br>58:01	C1R.A*02:01, C1R.B*15:01, C1R.B*58:01;<br>9053 B-LCL (A*33:03, _B*44:03_)	C1R.parental;<br>C1R.B*44:03
16				AP015	03:01	40:06,<br>58:01	C1R.A*03:01, C1R.B*58:01;<br>A21 B-LCL (_A2_, B40)	C1R.parental;<br>C1R.A*02:01

[a]A or B denotes to serological HLA typing, A* or B* denotes 4-digit high resolution genomic HLA typing. [#]Background IFNγ responses to C1R.parental were subtracted from responses to C1R transfectants; For B-LCL expressing multiple HLA-A/B, responses to alleles of interest were isolated by subtraction of IFNγ responses to C1R transfectants expressing the alternate alleles (in *italics*).
