# Supplementary material for: HLA-B57 micropolymorphism defines the sequence and conformational breadth of the immunopeptidome
Source: Nat Commun. 2018 Nov 8;9:4693. doi: 10.1038/s41467-018-07109-w (PMC6224591; doi:10.1038/s41467-018-07109-w)
Supplement: Supplementary file 2 — Description of Additional Supplementary Files [file 41467_2018_7109_MOESM2_ESM.pdf]

Legends for supplementary data files:

Supplementary Data 1: Pooled lists of HLA-B*57:01, HLA-B*57:03 and HLA-B*58:01 ligands.

Peptides identified in three replicate experiments by LC-MS/MS with a confidence greater than that for a 5% FDR and used to describe the peptide-binding motifs.

Supplementary Data 2: Peptide repertoire overlap of HLA-B*57:01, HLA-B*57:03 and HLA-B*58:01.

Peptide repertoire overlap of HLA-B*57:01, HLA-B*57:03 and HLA-B*58:01 as identified by LC-MS/MS. During comparison, if a peptide was identified in a data set with a confidence greater than or equal to 95 it was considered valid. Equally if a peptide was sequenced in a data set with a confidence between 20 and 95, but it appeared in another data set with confidence greater than or equal to 95, it was considered common to both data sets. Modifications were considered in the comparison of peptide identifications (Sheet 1, Seq&Mod), and comparisons based on sequence alone are calculated from these identifications in Sheet 2(Sequence only).

Supplementary Data 3: List of peptide contaminants disregarded from analyses.

Reason for removal is noted as common contaminant (common contaminant in similar experiments), C1R parental (observed in similar HLA isolation from C1R parental cells), HLA (peptides derived from HLA molecules) and HLA-C*04:01 motif (possessing anchor residues at P2 [F/Y] and P3 [D] or P2 and PΩ [LFVM] consistent with ligands of endogenous HLA-C*04:01 of C1R cells).
