# Supplementary material for: HLA-B57 micropolymorphism defines the sequence and conformational breadth of the immunopeptidome
Source: Nat Commun. 2018 Nov 8;9:4693. doi: 10.1038/s41467-018-07109-w (PMC6224591; doi:10.1038/s41467-018-07109-w)
Supplement: Supplementary file 6 — Reporting Summary [file 41467_2018_7109_MOESM6_ESM.pdf]

nature research

Corresponding author(s): Anthony Purcell and Julian Vivian

Reporting Summary

Nature Research wishes to improve the reproducibility of the work that we publish. This form provides structure for consistency and transparency in reporting. For further information on Nature Research policies, see Authors & Referees and the Editorial Policy Checklist.

Statistical parameters

When statistical analyses are reported, confirm that the following items are present in the relevant location (e.g. figure legend, table legend, main text, or Methods section).

n/a	Confirmed	
☐	☒	The exact sample size (*n*) for each experimental group/condition, given as a discrete number and unit of measurement
☐	☒	An indication of whether measurements were taken from distinct samples or whether the same sample was measured repeatedly
☐	☒	The statistical test(s) used AND whether they are one- or two-sided<br>*Only common tests should be described solely by name; describe more complex techniques in the Methods section.*
☒	☐	A description of all covariates tested
☒	☐	A description of any assumptions or corrections, such as tests of normality and adjustment for multiple comparisons
☒	☐	A full description of the statistics including central tendency (e.g. means) or other basic estimates (e.g. regression coefficient) AND variation (e.g. standard deviation) or associated estimates of uncertainty (e.g. confidence intervals)
☒	☐	For null hypothesis testing, the test statistic (e.g. *F*, *t*, *r*) with confidence intervals, effect sizes, degrees of freedom and *P* value noted<br>*Give P values as exact values whenever suitable.*
☒	☐	For Bayesian analysis, information on the choice of priors and Markov chain Monte Carlo settings
☒	☐	For hierarchical and complex designs, identification of the appropriate level for tests and full reporting of outcomes
☒	☐	Estimates of effect sizes (e.g. Cohen's *d*, Pearson's *r*), indicating how they were calculated
☐	☒	Clearly defined error bars<br>*State explicitly what error bars represent (e.g. SD, SE, CI)*

Our web collection on statistics for biologists may be useful.

Software and code

Policy information about availability of computer code

Data collection	Software are described in the methods and include:<br>FACSDIVA TM (BD Biosciences); Analyst 1.6.2 (SCIEX, USA); Unicorn 5.11
Data analysis	Commercial and open source:<br>FlowJo software vs8-10 (Tree Star Inc., USA); ProteinPilotTM software version 5.0 (SCIEX, USA); iceLogo v1.2; Skyline 64bit 3.5.0.9319 (MacCoss Laboratory); MOSFLM 1.0.7 and SCALA 3.3.2 from the CCP4 program suite; PHASER 2.5.1; COOT 0.7; PHENIX 1.9; MOLPROBITY 4.4; Graphpad Prism v7; Microsoft Excel;<br>Custom:<br>https://github.com/jlmendozabio/covariation_stats<br>https://github.com/ParhamLab/PeptidePCA/tree/master/R

For manuscripts utilizing custom algorithms or software that are central to the research but not yet described in published literature, software must be made available to editors/reviewers upon request. We strongly encourage code deposition in a community repository (e.g. GitHub). See the Nature Research guidelines for submitting code & software for further information.

Data

Policy information about availability of data

All manuscripts must include a data availability statement. This statement should provide the following information, where applicable:

- Accession codes, unique identifiers, or web links for publicly available datasets
- A list of figures that have associated raw data
- A description of any restrictions on data availability

Proteomics datasets analysed during this study have been deposited to the ProteomeXchange Consortium via the PRIDE70 partner repository with the dataset identifiers PXD008570 [https://www.ebi.ac.uk/pride/archive/projects/PXD008570] (C1R.B*57:01 LC-MS/MS), PXD008571 [https://www.ebi.ac.uk/pride/archive/projects/PXD008571] (C1R.B*57:03 LC-MS/MS), PXD008572 [https://www.ebi.ac.uk/pride/archive/projects/PXD008572] (C1R-B*58:01 LC-MS/MS) and PXD009850 [https://www.ebi.ac.uk/pride/archive/projects/PXD009850] (LC-MRM). Coordinates and structure factors were deposited in the PDB with the following codes: B5701-LSSPVTKSW 5VUD [http://dx.doi.org/10.2210/pdb5VUD/pdb]; B5701-LTVQVARVW 5VUE [http://dx.doi.org/10.2210/pdb5VUE/pdb]; B5701- LTVQVARVY 5VUF [http://dx.doi.org/10.2210/pdb5VUF/pdb]; B5703-LSSPVTKSW 5VVP [http://dx.doi.org/10.2210/pdb5VVP/pdb]; B5703-LTVQVARVW 5VWD [http://dx.doi.org/10.2210/pdb5VWD/pdb]; B5703-LTVQVARVY 5VWF [http://dx.doi.org/10.2210/pdb5VWF/pdb]; B5801-LSSPVTKSW 5VWH [http://dx.doi.org/10.2210/pdb5VWH/pdb]; B5801-LTVQVARVW 5VWJ [http://dx.doi.org/10.2210/pdb5VWJ/pdb]. All other data is available from the corresponding author on reasonable request.

Field-specific reporting

Please select the best fit for your research. If you are not sure, read the appropriate sections before making your selection.

☒ Life sciences ☐ Behavioural & social sciences ☐ Ecological, evolutionary & environmental sciences

For a reference copy of the document with all sections, see nature.com/authors/policies/ReportingSummary-flat.pdf

Life sciences study design

All studies must disclose on these points even when the disclosure is negative.

Sample size	No sample size calculation was performed, sample number for mixed lymphocyte reactions (MLR) was determined by sample availability. Experiments were performed to demonstrate the capacity for alloreactivity between HLA-B*57:01 and HLA-B*58:01, which is evident even with this small number of individuals.
Data exclusions	All collected MLR data was included
Replication	16 independent MLRs were performed
Randomization	Samples were allocated to different groups based on their genotype for the HLA-B locus
Blinding	Blinding was not possible as knowledge of HLA expression was necessary for correct performance of the assay

Reporting for specific materials, systems and methods

Materials & experimental systems

n/a	Involved in the study
☐	☒ Unique biological materials
☐	☒ Antibodies
☐	☒ Eukaryotic cell lines
☒	☐ Palaeontology
☒	☐ Animals and other organisms
☐	☒ Human research participants

Methods

n/a	Involved in the study
☒	☐ ChIP-seq
☐	☒ Flow cytometry
☒	☐ MRI-based neuroimaging

Unique biological materials

Policy information about availability of materials

Obtaining unique materials	Blood samples from healthy donors were provided with informed consent, the availability of which is subject to regulation for the use of human samples

Antibodies

Antibodies used	W6/32<br>Goat F(ab')2 Anti-Mouse IgG(H+L), Human ads-PE<br>anti-CD8 PerCP-Cy5.5 (clone SK1, catalogue number 341051, Becton Dickinson [BD] Biosciences, USA)<br>anti-CD4 PE (clone RPA-T4, catalogue number 555347, BD Biosciences)<br>anti-IFNg PE-Cy7 (1:250, clone B27, catalogue number 557643, BD Biosciences, USA
Validation	W6/32: In house staining comparison of HLA class I low (C1R) vs C1R transfected with HLA class I molecules<br>anti-CD8 PerCP-Cy5.5 (clone SK1, catalogue number 341051) - validation available from manufacturer: http://www.bdbiosciences.com/external_files/Doc_Recon_2.0/is/tds/23-1301.pdf<br>anti-CD4 PE (clone RPA-T4, catalogue number 555347, BD Biosciences) - validation available from manufacturer: http://www.bdbiosciences.com/external_files/Doc_Recon_2.0/pm/tds/555347.pdf<br>anti-IFNg PE-Cy7 (1:250, clone B27, catalogue number 557643) - validation available from manufacturer: http://www.bdbiosciences.com/external_files/Doc_Recon_2.0/pm/tds/557643.pdf

Eukaryotic cell lines

Policy information about cell lines

Cell line source(s)	C1R transfectants were produced within the McCluskey laboratory (Peter Doherty Institute, University of Melbourne, Victoria), T241, A21 were provided by the Victorian Transplantation and Immunogenetics Service (West Melbourne, Victoria).
Authentication	Increased HLA class I expression (as compared to C1R parental) was confirmed via flow cytometry after staining with the HLA class I pan specific monoclonal antibody W6/32 (produced in house from the W6/32 hybridoma) and Phycoerythrin Goat Anti-mouse IgG (Multiple Absorption) (1:500, Southern Biotech, USA).
Mycoplasma contamination	All cell lines tested negative for mycoplasma contamination
Commonly misidentified lines<br>(See ICLAC register)	No commonly misidentified cell lines were used

Human research participants

Policy information about studies involving human research participants

Population characteristics	At least 2 digit HLA typing at the HLA-A and HLA-B loci were the only variables considered.
Recruitment	Participants were volunteers recruited either through the Australian Bone Marrow Donor registry, or at Monash University who have a willingness to donate blood for research purposes.

Flow Cytometry

Plots

Confirm that:

- [x] The axis labels state the marker and fluorochrome used (e.g. CD4-FITC).
- [x] The axis scales are clearly visible. Include numbers along axes only for bottom left plot of group (a 'group' is an analysis of identical markers).
- [x] All plots are contour plots with outliers or pseudocolor plots.
- [ ] A numerical value for number of cells or percentage (with statistics) is provided.

Methodology

Sample preparation	PBMC were isolated using Ficoll–Paque (GE Healthcare, Sweden) and density gradient centrifugation. T cell cultures were generated from 5 x 10^6 responder PBMC stimulated with 2.5 x 10^6 irradiated allogeneic PBMC. Culture medium was supplemented with 20 U mL-1 recombinant human IL-2 (Cetus) and changed every 2-3 days to maintain saturating levels of nutrients and growth factors. On day 13, 2 x 10^5 responders from the T cell culture were restimulated with 105 B-LCLs expressing allo-HLA. After 2 hours of coincubation (37 °C, 5 % CO2), 10 µg mL-1 Brefeldin A (Sigma-Aldrich, USA) was added for a further 4 hours. Responder CD8+ T cells were stained with anti-CD8 PerCP-Cy5.5 (1:20, clone SK1, catalogue number 341051, Becton Dickinson [BD] Biosciences, USA), anti-CD4 PE (1:20, clone RPA-T4, catalogue number 555347, BD Biosciences) and a viability dye (1:750, LIVE/DEAD™ Fixable Aqua Dead Cell Stain, 405nm excitation, catalogue number L34957, Thermo Fisher), fixed with 1 % paraformaldehyde (ProSciTech, Australia) and permeabilised with 0.3 % Saponin (Sigma-Aldrich, USA) containing anti-IFN-g PE-Cy7 (1:250, clone B27, catalogue number 557643, BD Biosciences, USA)
Instrument	LSRII flow cytometer (BD, USA)
Software	collection: BD FACSDIVA™ software; analysis: using FlowJo software (Tree Star Inc., USA)

Cell population abundance	No sorting was performed
Gating strategy	Lymphocytes were gated on forward scatter (FSC) and side scatter (SSC), and single cells gated using FSC-width (FSC-W) compared to FSC-height (FSC-H) then SSC-W and SSC-H. Viable cells isolated at those negative for staining with LIVE/DEAD™ Fixable Aqua Dead Cell Stain. Live T cells were phenotyped based on CD4 and CD8 expression (d) and the CD8+, CD4- quadrant selected for analysis of IFNγ expression.

☒ Tick this box to confirm that a figure exemplifying the gating strategy is provided in the Supplementary Information.
